# Identification and Estimation of the Average Causal Effects Under Dietary Substitution Strategies

**DOI:** 10.1002/sim.70007

**Published:** 2025-02-20

**Authors:** Yu‐Han Chiu, Lan Wen

**Affiliations:** ^1^ Department of Public Health Sciences Penn State College of Medicine Hershey Pennsylvania USA; ^2^ CAUSALab and Department of Epidemiology Harvard T.H. Chan School of Public Health Boston Massachusetts USA; ^3^ Department of Statistics and Actuarial Science University of Waterloo Waterloo Ontario Canada

**Keywords:** causal effects, double robustness, influence functions, substitution effects, targeted maximum likelihood estimation

## Abstract

The 2020–2025 Dietary Guidelines suggest that most people can improve their diet by making some changes to what they eat and drink. In many cases, these changes involve simple substitutions. For instance, the Dietary Guidelines recommend choosing chicken instead of processed red meat to reduce sodium intake and switching from refined grains to whole grains to increase dietary fiber intake. The question about such dietary substitution strategies seeks to estimate the average counterfactual outcome under a hypothetical intervention that replaces a food an individual would have consumed in the absence of intervention with a healthier substitute. In this work, we will show the conditions under which the average causal effects of substitution strategies can be non‐parametrically identified, and provide efficient estimators for our proposed dietary substitution strategies. We evaluate the performance of our proposed methods via simulation studies and apply them to estimate the effect of substituting processed red meat with chicken on mortality, using data from the Nurses' Health Study.

## 1 Introduction

According to the 2020–2025 Dietary Guidelines, achieving a healthy dietary pattern will require changes in food and beverage choices. For most individuals, these changes can be made by simple substitutions. Typically, the substitute food is similar to the one being replaced, providing a sustainable way to improve dietary quality without changing one's entire diet. For example, processed red meat can be replaced with chicken, refined grains with whole grains, butter with olive oil, and whole milk with low‐fat milk. The emphasis on dietary substitution is based on the premise that if an individual were asked to reduce their intake of processed red meat, they would be more likely to increase the intake of other foods to compensate for reductions in processed red meat intake. Given that the average causal effect of substitution strategies varies depending on the replacement food that is selected, understanding the average causal effect of specific dietary substitutions is highly relevant for informing personal decisions and public health recommendations.

Studies on dietary substitution seek to estimate the average causal effect of replacing one food group with another on an outcome of interest. In the absence of randomized trials, substitution analyses have been widely used to address this question using data from observational cohorts in nutritional epidemiology [1, 2, 3, 4, 5, 6]. For example, to estimate the effect of replacing processed red meat with chicken, standard substitution analyses typically include all protein food groups (e.g., processed red meat, non‐processed red meat, chicken, low‐fat dairy, high‐fat dairy, legumes, and nuts), as well as total caloric intake in an outcome regression model. The difference in the estimated coefficients from the model are then used to quantify the effect of a one‐unit increase in chicken (i.e., the food substitute) for a corresponding one‐unit decrease in processed red meat (i.e., the food being substituted) while keeping all other variables at the same level. Despite the attempt to mimic an isocaloric dietary substitution by adjusting for total energy intake in the model, these studies have not explicitly considered the definition of their causal estimand nor the assumptions for identifying and consistently estimating the underlying average causal effect. Foremost, most studies have not taken into account an individual's natural dietary behaviors and explicitly considered a dietary strategy that could be reasonably implemented in the real world. For instance, the strategy of decreasing one serving of processed red meat and increasing one serving of chicken cannot be applied universally. Some individuals, such as those who do not consume any meat, will have a zero chance of following the strategy under consideration. Moreover, for individuals already consuming a substantial amount of chicken, further increasing their intake may exceed the range supported by the observational data. Furthermore, many existing studies rely on unrealistic parametric assumptions, such as linearity and absence of interaction terms, to simplify estimation and interpretation of the average causal effect. Specifically, these assumptions allow researchers to use parameters from the outcome regression model (i.e., the coefficients associated with the variables under intervention) as a direct estimate of the average causal effect of replacing one unit of the original food with one unit of the substitute. Finally, while most studies acknowledge the limitations of unmeasured confounding and measurement errors of dietary exposures when using observational data, sufficient assumptions for non‐parametric identification and estimation of the average causal effect of dietary substitution have not been formalized.

In this paper, we sketch a framework for estimating the average causal effect of dietary substitution strategies using observational data under a point exposure setting. As we elaborate further below, our dietary strategies of substitution are similar to a type of intervention that can depend on the natural value of exposure [7, 8, 9, 10, 11]. To address the intricacies of dietary exposure, we present a novel theoretical and methodological framework specifically designed for complex, multiple‐exposure settings. The rest of the article is structured in the following manner. In Section 2, we present a motivating example of a target trial that would answer our causal question regarding the dietary substitution of processed red meat with chicken, which serves as a reference point throughout. In Section 3, we introduce our notations and causal model, describe the data structure available in the observational studies, and define causal estimands under our proposed dietary substitution strategies. In Section 4, we outline the assumptions sufficient for the identification of the causal estimand, and in Section 5, we propose efficient estimators for our causal estimand. In Section 6, we assess the performance of our proposed estimators in simulation studies, and in Section 7, we implement our proposed estimators to estimate the effect of dietary substitution strategies using data from a large observational cohort study. We conclude with some discussions on the practical implications of dietary substitution strategies in Section 8.

## 2 Motivating Example: Dietary Substitution Strategies and Mortality Risk

To fix ideas, consider interest in estimating the average causal effect of substituting processed red meat with equal servings of chicken on an outcome of interest at the end of a study period. Ideally, a well‐designed randomized controlled trial would provide evidence to address this question. The target population of the trial would include individuals aged 60 and above with no prior diagnosis of diabetes, cardiovascular disease, or cancer. Individuals with severe medical diseases (e.g., Parkinson's disease, amyotrophic lateral sclerosis, multiple sclerosis, ulcerative colitis) or those with extreme obesity (a body mass index equal to or exceeding $40\;\text{kg}/m^{2}$) would be excluded because they may be less likely to follow the substitution strategy. The primary outcome is 2‐year all‐cause mortality, with follow‐up starting from the time of randomized assignment until death, loss to follow‐up, or administrative end of follow‐up, whichever occurs first. Eligible individuals who report their baseline characteristics, diet, and risk factors will be randomized to one of the following dietary strategies:

1. Maintain the regular diet as you would have in the absence of intervention.
2. Maintain the regular diet as you would have in the absence of intervention, except that replace all servings of processed red meat you would eat (in the absence of intervention) with equivalent servings of chicken.

To be more precise, Strategy 2 requires participants to first determine what they would eat, including processed red meat, chicken, and other foods, in the absence of any intervention. They are then instructed to replace processed red meat with equivalent servings of chicken while keeping the intake of other foods unchanged. Such dietary strategies may involve a point intervention, where the intervention occurs once, either at or shortly after the start of the follow‐up period. More commonly, however, dietary studies involve sustained strategies, where interventions occur over multiple time points over the follow‐up [12]. It is important to note that, regardless of point or sustained interventions, once individuals are assigned to the substitution strategy, their natural intake during the study period (i.e., the amount of processed red meat and chicken that they would eat in the absence of intervention) will not be observed. This poses challenges in executing the substitution strategy and objectively evaluating individuals' adherence to it.

To address these challenges, it is imperative to collect individuals' planned or intended dietary intake right before the intervention and posit that their reported planned intake levels will equal what they would have eaten in the absence of intervention. In a real randomized controlled trial, the implementation of such an intervention may proceed as follows: Participants would receive a dietary training session before the intervention. In this dietary session, participants would be asked about what they anticipate consuming during the intervention period if their diet was not intervened upon. Under Strategy 2, if an individual planned to consume, for example, two servings of processed red meat and one serving of chicken (hereafter referred to as the *planned or intended intake*), then they would be instructed to eat three servings of chicken and zero processed red meat.

To assess adherence to the assigned dietary strategy, participants would also complete a questionnaire on what they actually ate (hereafter referred to as the *actual intake*) over the intervention period. For a short‐term trial with a point intervention, individuals' planned and actual intake may only be collected once. For a long‐term trial with a sustained intervention, individuals' planned and actual intake may be collected more frequently during the study period as diet may vary over a longer time span. See Supporting Information S1: Appendix A for an illustration of a timeline for collecting the planned intake and actual intake in an idealized hypothetical trial. Upon completion of this trial, researchers can estimate different causal effects, such as the average causal effect of randomized assignment (an intention‐to‐treat effect) and the average causal effect had everyone adhered to their assigned strategy over the study period (a per‐protocol effect; see [13] for more precise definitions).

In the absence of a randomized controlled trial, we aim to estimate the average causal effect of the above substitution strategies using data from the Nurses' Health Study (NHS). The NHS is a prospective cohort study established in 1976 with an enrollment of more than 120 000 female registered nurses aged 30 to 55 from the United States at its inception [14, 15]. Information on various lifestyle factors and newly diagnosed diseases is updated every 2 years via questionnaires, with new disease diagnoses confirmed via medical records and deaths identified using the National Death Index. Additionally, dietary assessments are collected every 4 years via a validated food frequency questionnaire (FFQ; [16, 17, 18, 19, 20, 21]). The NHS provided groundbreaking evidence indicating that foods with trans fat pose particular harm [22], which drove a significant shift in nutrition policy in the past decade [23]. To focus on the formalization of our causal framework and align with prior work on the interpretation of substitution analyses, we consider a point intervention setting and define baseline (i.e., time zero) as the date of return of the 2010 questionnaire. Since there is no assignment or an appropriate assignment analog in the NHS, under a point intervention setting, the causal estimand of interest in the observational emulation would be the average causal effect of following the substitution strategy at baseline on 2‐year mortality. If we are willing to assume dietary behaviors remain unchanged or stable over a certain period represented by an FFQ at baseline (e.g., 1 year), then our causal estimand can be interpreted as the effect of adhering to the substitution strategy for that time frame on 2‐year mortality. In the next sections, we introduce some notation and describe how to estimate the average causal effect of dietary substitution strategies using the available observational data from NHS.

## 3 Causal Models and Estimands

### 3.1 Notation and Basic Setup

Denote $\left(O_{1},\ldots ,O_{n}\right)$ as the baseline sample of independent and identically distributed individuals who meet the eligibility criteria for the above target trial.

Under a point intervention setting, we assume dietary behaviors remain unchanged over the 1 year following the baseline measurement. This assumption is supported by previous validation studies showing a high degree of reproducibility for nutrient intakes assessed by FFQs administered 1 year apart [16, 21].

For simplicity and without loss of generality, let 𝒜 denote the food group (e.g., processed red meat) that we would like to substitute food group ℬ (e.g., chicken) with, and let 𝒞 denote all other food groups not involved in substitution (e.g., fish, dairy, legumes, vegetables, etc.). We let Ã, $\tilde{B}$, and $\tilde{C}$ denote the weekly servings of food intake that participants *plan* (or *intend*) to consume in groups 𝒜, ℬ, and 𝒞, respectively, immediately before the intervention. For example, Ã denotes planned intake for processed red meat in absence of intervention. Let A, B, and C denote the weekly servings of food intake that participants *actually* consume in groups 𝒜, ℬ, and 𝒞, respectively. Let Y denote a binary outcome (e.g., mortality) by the end of a specified study period, L a set of potential baseline confounders (e.g., physical activity, body size, age, sex) that affect both baseline planned dietary intake and outcome, and U a set of potential baseline factors influencing planned dietary intake (e.g., dietary preference). We assume the temporal ordering for variables compiled in a vector V, namely: $V=(U,L,Ã,\tilde{B},\tilde{C},A,B,C,Y)$, where V contains both the unobserved variable U and the observed variables from the distribution P given by $O=(L,Ã,\tilde{B},\tilde{C},A,B,C,Y)∼P$.

### 3.2 Causal Models and Causal Estimand

**FIGURE 1:**
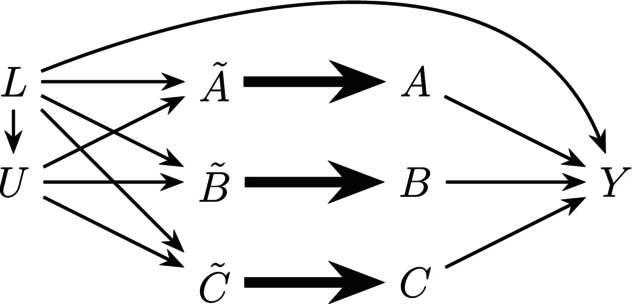
A simplified Directed Acyclic Graph (DAG) depicting a data structure of the observational data. Here, Ã, $\tilde{B}$, and $\tilde{C}$ denote the planned weekly portions of food intake in groups 𝒜, ℬ and 𝒞; A, B, and C denote the actual weekly portions of food intake in groups 𝒜, ℬ and 𝒞; Y is a binary outcome; L is a set of potential baseline confounders that affect planned dietary intake and outcome; and U is a set of potential baseline common causes of planned dietary intake decisions. Bold arrows are used to emphasize equivalence relationships assumed herein.

Throughout, we operate under the assumption that a causal Directed Acyclic Graph (DAG) depicts the Finest Fully Randomized Causally Interpreted Structural Tree Graph (FFRCISTG) model [10, 24, 25]. The causal DAG in Figure 1 depicts a simple structure for our running example (see Supporting Information S1: Appendix A for a DAG under a more complex and realistic setting). We note that in DAG (Figure 1), the lack of arrows between $Ã,\tilde{B}$ and $\tilde{C}$ depicts the assumption that dietary decisions only depend on underlying latent characteristics (e.g., food preference), such that conditional on these latent factors, Ã, $\tilde{B}$ and $\tilde{C}$ are independent. DAG (Figure 1) also encodes the assumption that in the absence of intervention, an individual's planned dietary intake will equal their actual dietary intake, which we formalize in Section 4.

We use Single World Intervention Graphs (SWIGs) to represent the joint intervention on multiple exposures. Informally, a SWIG can be viewed as a causal DAG depicting a counterfactual world where the values of exposure variables are set in accordance with a specific hypothetical intervention. Let g denote a dietary strategy or intervention rule that specifies how intake levels of exposure variables are intervened upon. We use the notation $Y^{g}$ for a counterfactual outcome under a dietary strategy g. Following Richardson and Robins [10], we use $X^{g^{†}}$ to denote *manipulated exposure variable* under g, i.e., the value that exposure takes under strategy g, where X represents an exposure variable that we are intervening on (e.g., X could be A, B and/or C in Figure 1). In contrast, $X^{g}$ denotes *natural exposure variable* under g, i.e., the value that variable X takes in a hypothetical world in which the intervention rule g is stopped just before intervening on X. For a point intervention, $X^{g}$ is simply defined as the value that exposure X takes in the absence of intervention (i.e., the observed exposure variable at baseline). In our dietary substitution strategy g that we formally define below, we refer to $X^{g^{†}}$ as the *intervened intake* of X, and $X^{g}$ as the *actual intake* of X in absence of intervention. Next, we consider a dietary substitution strategy that is relevant to our motivating example.

Consider a substitution strategy g where the weekly servings of processed red meat (𝒜) are replaced with that of chicken (ℬ).

g: If an individual plans to eat a servings of 𝒜 and b servings of ℬ per week, then ensure they eat 0 serving of 𝒜 and a+b servings of ℬ. The intake of 𝒞 should remain unchanged from their intended levels.

Since the counterfactual outcome may differ depending on the interventions on other food groups (𝒞), strategy g also specifies what to eat for those other food groups to ensure a well‐defined intervention [26]. Moreover, to avoid potential positivity violations that may arise when individuals already consuming significant amounts of chicken need to further increase their intake beyond the range supported by our data, we can refine our strategy by specifying a threshold for $B^{g^{†}}$, denoted as x. More specifically, x can often be defined as max{supp(B)}. Alternatively, it may be specified a priori as a level less than max{supp(B)} to ensure that an individual's consumption of ℬ does not exceed acceptable limits, based on subject matter knowledge. The modified dietary substitution intervention g is now defined as follows:

(1) $$\begin{array}{ll} & \;g:\text{If an individual plans to eat}\;a\;\text{servings of}𝒜\;\text{and}\;b\;\\ & \;\text{servings of}\;ℬ,\;\text{then ensure they replace processed red}\\ & \;\text{meat with chicken, such that they}\text{eat}\;0\;\text{serving of processed}\\ & \;\text{red meat}\text{and}\;\max(x,a+b)\;\text{servings of}\;ℬ.\;\text{The}\text{intake}\text{of}\;𝒞\;\\ & \;\text{should}\text{remain unchanged from their intended levels.} \end{array}$$

**FIGURE 2:**
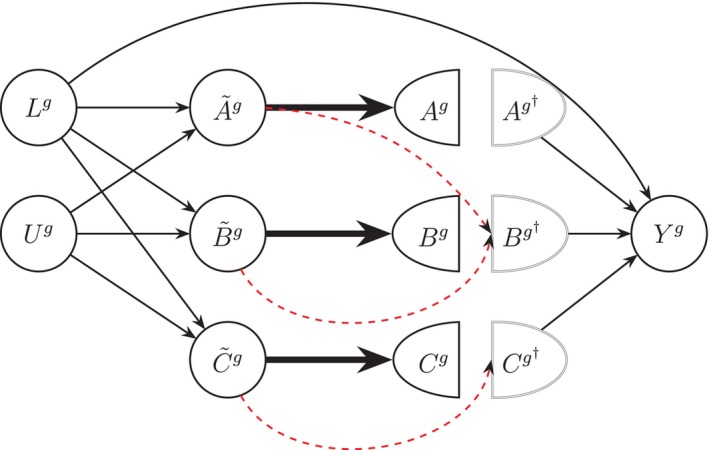
Single World Intervention Graph (SWIG) depicting intervention g described in Section 3. Bold arrows are used to emphasize equivalence relationships assumed herein.

See Figure 2 for a corresponding SWIG under this intervention. We are interested in the average counterfactual outcome of a specified dietary substitution intervention g in a target population, denoted by $\mu^{g}=\mu^{g}(P):=E(Y^{g})$, where $Y^{g}=Y^{a,b,c}$ and (a,b,c) are values within the support of $\left(A^{g^{†}},B^{g^{†}},C^{g^{†}}\right)$. The causal contrast of interest in the target population is the average causal effect defined as the difference in (1) the average outcome in the observed data (i.e., under no intervention on diet intake) and (2) the average counterfactual outcomes under dietary strategy g, i.e., $\Delta =E(Y)-E(Y^{g})$. Here, Δ quantifies the reduction in the 2‐year risk of all‐cause mortality had all participants followed the substitution strategy. In the next section, we focus our attention on identifying $E(Y^{g})$ under a set of causal assumptions.

## 4 Identifiability Assumptions and Identification

In this section, we give sufficient assumptions and identifying formula of a causal parameter $E(Y^{g})$ for a dietary substitution strategy g where under this strategy, $A^{g^{†}}$ is set to 0, $B^{g^{†}}$ is defined by a deterministic function $d(Ã,\tilde{B})$ and $C^{g^{†}}$ is set to the same level as baseline (i.e., $\tilde{C}$ ) in the observed data. In formal terms, this is similar to interventions that can depend on the natural value of exposure. Robins et al. [7] first defined interventions of this type and was later adopted and further studied in the works of [8], [9], [10], and [11]. These interventions are also referred to as “modified treatment policies” in literature [9, 11]. Unlike existing literature, our setting is more complex because our intervention involves multiple exposures wherein the intervened value of one exposure may depend on the intended values of several exposure variables. Note that Young et al. [27] also consider interventions on an exposure that depends on one's intention, though they focused solely on a single exposure variable. To formally state our identifiability conditions, we invoke the following set of assumptions:

Assumption 1 (Consistency) If A=a, B=b and C=c, then $Y^{a,b,c}=Y$.

Assumption 2 (Determinism) $Ã=A,\;\;\tilde{B}=B,\;\;\tilde{C}=C$ (with probability 1)

The consistency assumption ensures that the exposures are well defined and that any means of arriving at a particular exposure level for (a,b,c) results in the same outcome $Y^{a,b,c}$ [26, 28, 29]. Ideally, planned intake is collected right before actual intake. However, since observational studies often only collect what people actually eat in a coarsened time frame, we assume in the determinism assumption that the planned intake level that participants would have reported is equal to their subsequent actual intake in the absence of intervention.

Assumption 3 (Exchangeability) $Y^{a,b,c}⊥⊥(A,B)|(C,L)$,

Assumption 4 (Positivity)

$$\begin{array}{ll} \text{If}\;(0,b^{†},c,l) & \in \;\text{supp}(A^{g^{†}},B^{g^{†}},C,L),\text{then}\;(0,b^{†},c,l)\\ & \in \;\text{supp}(A,B,C,L). \end{array}$$

where supp(X) is the support of a random variable X in the target population. The exchangeability assumption states that within strata defined by the actual intake C and baseline covariates L, there are no unmeasured common causes between actual intake variables (A,B) and outcome, and implies that planned intake has no direct effect on the outcome except through actual dietary intake. Finally, the positivity condition asserts that if within the target population there exists a non‐zero probability of finding subjects with characteristic (C,L)=(c,l), and who would have $\left(A^{g^{†}},B^{g^{†}})=(0,b^{†}\right)$ under the intervention, then there must also be a non‐zero probability of finding subjects within the same population with the same characteristic (C,L)=(c,l), who would have $\left(A,B)=(0,b^{†}\right)$ in the absence of intervention. The positivity condition also implies that within every possible level of an observed stratum of (C,L), there are always some individuals who have A=0.

Theorem 1 *Under Assumptions* *1*, *2*, *3*, *4*, *the counterfactual outcome under the* 𝒜 *‐for‐* ℬ *dietary substitution strategy* g, *is given by*:

(2) $$\mu^{g}=\underset{a,b,c,l}{\sum }E(Y|A=0,B=d(a,b),C=c,L=l)p(a,b,c|l)p(l)$$

where d(a,b) for Ã=a and $\tilde{B}=b$ in (2) is defined as follows:

(3) $$\begin{array}{l} d(a,b)=\left\{\begin{array}{ll} a+b,\; & \text{if}\;a+b\leq x\\ x,\; & \text{if}\;a+b>x \end{array}\right. \end{array}$$

Proof of identification can be found in Supporting Information S1: Appendix B. Under (3), we can define an intervened exposure distribution $q(b^{†}|\cdot )$ of $B^{g^{†}}$ as:

$$\begin{array}{ll} q(b^{†}|a,b,c,l) & =I(b^{†}=a+b)I(a+b\leq x)\\ & \;+I(b^{†}=x)I(a+b>x) \end{array}$$

That is, if an individual plans to eat a servings of 𝒜 and b servings of ℬ, and sum of a and b is no greater than x, then ensure they eat a+b servings of ℬ; otherwise, eat x servings of ℬ. As we show in Supporting Information S1: Appendix B,

$$\begin{array}{ll} \mu^{g}:=E(Y^{g}) & =\underset{a,b,c,l,b^{†}}{\sum }E(Y|A=0,B=d(a,b),\\ & \;C=c,L=l)p(a,b,c|l)p(l)\\ & =\underset{a,b,c,l,b^{†}}{\sum }E(Y|A=0,B=b^{†},\\ & \;C=c,L=l)q(b^{†}|a,b,c,l)p(a,b|c,l)p(c|l)p(l)\\ & =\underset{c,l,b^{†}}{\sum }E(Y|A=0,B=b^{†},\\ & \;C=c,L=l)\tilde{q}(b^{†}|c,l)p(c|l)p(l) \end{array}$$

where

$$\begin{array}{ll} \tilde{q}(b^{†}|c,l) & =\underset{0\leq a\leq b^{†}}{\sum }\{I(b^{†}\leq x)p_{B}(b^{†}-a|a,c,l)p_{A}(a|c,l)\\ & \;+\underset{a\geq 0}{\sum }I(b^{†}=x)\left[1-F_{B}(x-a|a,c,l)\right]\}p_{A}(a|c,l)\\ & =\underset{a\geq 0}{\sum }\{I(a\leq b^{†}\leq x)p_{B}(b^{†}-a|a,c,l)\\ & \;+I(b^{†}=x)\left[1-F_{B}(x-a|a,c,l)\right]\}p_{A}(a|c,l) \end{array}$$

and $F_{B}(x-a|a,c,l)=P(B\leq x-a|a,c,l)$. The summation given by $\sum_{0\leq a\leq b^{†}}$ in the first line comes from the fact that the support of B is 0 or greater, and so when $b^{†}=a+b$ under the intervention, this implies that $0\leq a\leq b^{†}$. As such, after rewriting $b^{†}$ as b, the inverse probability weighted (IPW) representation of the identifying formula is given by:

(4) $$E\left\{Y\frac{I(A=0)\tilde{q}(B|C,L)}{p(A|C,L)p(B|A,C,L)}\right\}$$

where

$$\begin{array}{ll} \tilde{q}(B|C,L) & :=\left[\underset{a}{\sum }\left\{I(a\leq B\leq x)p_{B}(B-a|a,C,L)\right.\right.\\ & \left.\;+\;I(B=x)\left[1-F_{B}(x-a|a,C,L)\right]\right\}p_{A}(a|C,L)] \end{array}$$

We also consider a comparable substitution strategy for scenarios where investigators aim to cap intake of ℬ at a maximum value x, while ensuring that the total combined intake of 𝒜 and ℬ remains unchanged before and after substitution. In Supporting Information S1: Appendix B, we illustrate how we can identify the average counterfactual outcome using observational data under this alternative strategy.

## 5 Estimation

### 5.1 Outcome Regression Estimation

Denote the estimate of the conditional mean outcome m(A,B,C,L):=m(A,B,C,L;P)=E(Y|A,B,C,L) as $\hat{m}(A,B,C,L):=m(A,B,C,L;\hat{P})$, which can be estimated parametrically with regression models or non‐parametrically using machine learning algorithms. The outcome regression model requires one to estimate $E(Y|A=0,B=B^{g^{†}},C,L)$ for all individuals using $\hat{m}(A,B,C,L)$. Continuing with the example given in (3), we note that $B^{g^{†}}$ equals d(a,b) when a+b≤x, given the individual's observed values of Ã and $\tilde{B}$ as a and b, respectively. Otherwise, if a+b>x, $B^{g^{†}}$ takes the value of x. The outcome regression estimator for $E(Y^{g})$ can be obtained by taking the average over the estimated conditional mean outcomes under the intervention g. That is,

$$\begin{array}{l} {\hat{\mu }}_{ORE}^{g}={\mathbb{P}}_{n}\{\hat{m}(0,B^{g^{†}},C,L)\} \end{array}$$

where we denote this estimator using ${\hat{\mu }}_{ORE}^{g}$ and define ${\mathbb{P}}_{n}(X)=\sum_{i=1}^{n}X_{i}$ for any variable X.

### 5.2 Inverse Probability Weighted Estimation

Denote the estimated distribution of exposure A conditional on exposure C and covariates L as ${\hat{\lambda }}_{a}(C,L):=\hat{P}(A=a|C,L)$, and denote the estimated distribution of B conditional on exposures A and C, and covariates L as ${\hat{\lambda }}_{b}(A,C,L):=\hat{P}(B=b|A,C,L)$. These distributions can be estimated parametrically using regression models or non‐parametrically using machine learning algorithms. An inverse probability weighted estimator for estimating $E(Y^{g})$ denoted by ${\hat{\mu }}_{IPW}^{g}$ can be obtained by solving the following set of estimating equations:

$$\begin{array}{l} 0={\mathbb{P}}_{n}\left\{Ŵ(Y-\mu_{IPW}^{g})\right\} \end{array}$$

where we define

$$\begin{array}{l} Ŵ=\frac{I(A=0)\hat{\tilde{q}}(B|C,L)}{{\hat{\lambda }}_{A}(C,L){\hat{\lambda }}_{B}(A,C,L)} \end{array}$$

and

$$\begin{array}{ll} \hat{\tilde{q}}(B|C,L) & =\left[\underset{a}{\sum }\left\{I(a\leq B\leq x){\hat{\lambda }}_{B-a}(a,C,L)\right.\right.\\ & \;\left.\left.+\;I(B=x)\left[1-\overset{x-a}{\underset{b=0}{\sum }}{\hat{\lambda }}_{b}(A,C,L)\right]\right\}{\hat{\lambda }}_{a}(C,L)\right] \end{array}$$

Here, ${\hat{\lambda }}_{A}(C,L)$ and ${\hat{\lambda }}_{B}(A,C,L)$ are defined as the estimated distributions of exposures A and B evaluated at random arguments, respectively.

### 5.3 Doubly Robust Estimation

Unlike $\mu_{IPW}^{g}$ and $\mu_{ORE}^{g}$, doubly robust estimators based on the efficient influence function for $E(Y^{g})$ can provide more robustness against model misspecification of the nuisance functions given by P(A=a|C,L), P(B=b|A,C,L) and E(Y|A,B,C,L).

Suppose that the observed data O follow a law P which is known to belong to $ℳ=\{P_{\theta }:\theta \in \Theta \}$, where Θ is the parameter space of θ. The efficient influence function $\varphi^{eff}(O)$ for a causal parameter $\mu^{g}\equiv \mu^{g}(\theta )$ in a non‐parametric model ${ℳ}_{\text{np}}$ that imposes no restrictions on the law of observed data O (other than positivity) is given by $d\mu^{g}(\theta_{t})/dt{|}_{t=0}=E\{\varphi^{eff}(O)S(O)\}$, where $d\mu^{g}(\theta_{t})/dt{|}_{t=0}$ is known as the pathwise derivative of the parameter $\mu^{g}$ along any parametric submodel of the observed data distribution indexed by t, and S(O) is the score function of the parametric submodel evaluated at t=0 [30, 31]. The efficient influence function for $\mu^{g}:=E(Y^{g})$ is given by:

(5) $$\begin{array}{ll} \varphi^{eff}(O) & =\frac{I(A=0)\tilde{q}(B|C,L)}{p(A|C,L)p(B|A,C,L)}\{Y-m(A,B,C,L)\}\\ & \;+m(0,B^{g^{†}},C,L)-\mu^{g} \end{array}$$

A proof is given in Supporting Information S1: Appendix C. This efficient influence function motivates the following doubly robust Targeted Maximum Likelihood Estimator (TMLE) (Algorithm 1):

ALGORITHM 1 **Algorithm for Targeted Maximum Likelihood Estimator (TMLE) of E(Y^g^).**

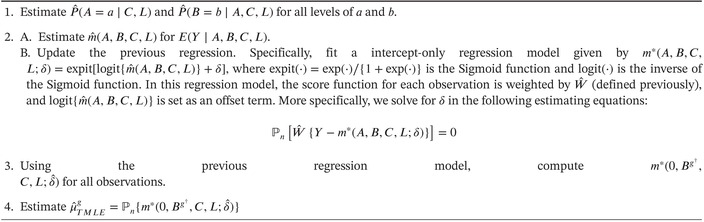

Theorem 2 (Weak convergence of TMLE) *Suppose that the conditions given in Supporting Information* S1: *Appendix D hold, and further suppose that the following condition also holds*:

$$\begin{array}{ll} & \left\|\hat{r}(A,B,C,L)-r(A,B,C,L)\|\;\|\hat{m}(A,B,C,L\right)\\ & \;-m(A,B,C,L)\|=o_{p}(n^{-1/2}) \end{array}$$

*where* $\|f(x){\|}_{2}={\left\{\int |f(x){|}^{2}dP(x)\right\}}^{1/2}$, *i.e., the* $L_{2}(P)$ *norm*, $r(A,B,C,L)=\frac{I(A=0)\tilde{q}(B|C,L)}{p(A,B|C,L)}$ *and* $\hat{r}(A,B,C,L)=\frac{I(A=0)\hat{\tilde{q}}(B|C,L)}{\hat{p}(A,B|C,L)}$. *Then*,

$$\begin{array}{l} \sqrt{n}\left({\hat{\mu }}_{TMLE}^{g}-\mu^{g}\right)⇝N(0,\sigma^{2}),\;\;\text{where}\;\sigma^{2}=\mathrm{Var}\{\varphi^{\text{eff}}(O)\}. \end{array}$$

We note here that the variance of TMLE can be estimated using the sandwich variance estimator or via bootstrap. As such, for any regular and asymptotically linear estimator ${\hat{\mu }}^{g}$ of $\mu^{g}$ in ${ℳ}_{np}$, it must be that $\sqrt{n}({\hat{\mu }}^{g}-\mu^{g})=n^{-1/2}\sum_{i=1}^{n}\varphi^{\text{eff}}(O_{i})+o_{p}(1)$. Furthermore, all regular and asymptotically linear estimators in ${ℳ}_{np}$ with efficient influence function equaling to $\varphi^{\text{eff}}(O)$ are asymptotically equivalent and attain the non‐parametric efficiency bound [32]. Based on the asymptotic property of ${\hat{\mu }}^{g}$, we can also specify the double robustness property of our estimator:

Corollary 1 *Under standard regularity conditions, the TMLE* ${\hat{\mu }}_{TMLE}^{g}$ *will be consistent and asymptotically normal under the union model* ${ℳ}_{union}={ℳ}_{1}\cup {ℳ}_{2}$ *where we define*:

*1*. *Model* ${ℳ}_{1}$: *working models for the joint distribution of* P(A=a,B=b|B,C,L) *are correctly specified*.
*2*. *Model* ${ℳ}_{2}$: *working model for* m(A,B,C,L) *is correctly specified*.

### 5.4 Marginal Structural Models

More generally, we can utilize marginal structural models (MSMs) to examine how the dependence of the average counterfactual outcome varies across different subpopulations of individuals defined by Z, a subvector of L (i.e., Z⊆L), or by a function of Z. We posit a working model $g(Z;\theta ):=\text{expit}\{s{\left(Z\right)}^{T}\theta \}$ that summarises this causal quantity, where g(Z;θ) is a known function of Z with unknown parameter vector $\theta \in R^{p}$, and s(Z) is a vector of linear predictors with the same dimensions as θ. For example, if p=2, then we could define $g(Z;\theta )=\text{expit}(\theta_{0}+\theta_{1}Z)$ whereby $s(Z)={\left(1,Z\right)}^{T}.$

In a broader sense, an MSM is often motivated by the question of interest and proposed by subject knowledge experts. Typically, the aim is to estimate parameters in a parsimonious MSM where the coefficients have simple interpretations. We can conceptualize the target statistical parameter defined by g(Z;θ) as a projection of the true causal quantity $E(Y^{g}|Z)$ onto this working model given by g(Z;θ). In simpler terms, we can perceive this projection as summarizing all possible conditional average counterfactual outcomes into a finite set of parameters. Following Petersen et al. [33] (see also [9, 34]), our causal quantity of interest for a binary outcome Y is defined as:

$$\begin{array}{ll} \theta (P) & =\underset{\theta \in R^{p}}{\text{arg min}}\\ & \;E[\xi (L;P)\log\{g(Z;\theta )\}+\{1-\xi (L;P)\}\log\{1-g(Z;\theta )\}] \end{array}$$

where $\xi (L;P)=E\left\{m(0,B^{g^{†}},C,L)|L\right\}=E(Y^{g}|L)$ and θ=θ(P) is the least false parameter value (or the true causal quantity under a correctly specified working model) that satisfy the following equation:

$$\begin{array}{l} 0=E\left[\frac{▿g(Z;\theta )}{g(Z;\theta )(1-g(Z;\theta ))}\left\{\xi (L;P)-g(Z;\theta )\right\}\right] \end{array}$$

where ▿g(Z;θ)=dg(Z;θ)/dθ.

Theorem 3 *Under* ${ℳ}_{np}$, *the efficient influence functions of the parameters* θ(P) *defined in the MSMs are characterized up to proportionality as follows*:

(6) $$\begin{array}{ll} \varphi_{msm}^{eff}(O) & \propto \;s(Z)\left[\frac{I(A=0)\tilde{q}(B|C,L)}{p(A|C,L)p(B|A,C,L)}\right.\\ & \;\{Y-m(A,B,C,L)\}+\left\{m(0,B^{g^{†}},C,L)-g(Z;\theta )\right\}] \end{array}$$

A TMLE for an MSM based on the efficient influence function given by (6) is given by the following algorithm 2:

ALGORITHM 2 **Algorithm for Targeted Maximum Likelihood Estimator (TMLE) of MSM.**

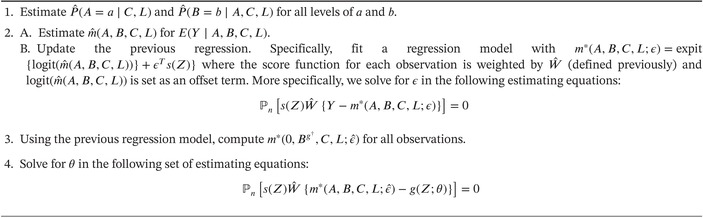

Theorem 4 (Weak convergence of TMLE for MSM) *Suppose that the conditions given in Supporting Information* S1: *Appendix D hold, and further suppose that the product mean‐squared error condition of Theorem* *2* *holds. Then*,

$$\begin{array}{l} \sqrt{n}\left(\hat{\theta }-\theta \right)⇝MVN\left(0,{\mathbb{C}}^{-1}\text{Cov}\{\phi (O;\eta ,\theta \}{\mathbb{C}}^{{-1}^{T}}\right) \end{array}$$

*where* $\eta =\eta (P):={\left(\eta_{1}(P),\eta_{2}(P)\right)}^{T}={\left(P(A,B|C,L),m(A,B,C,L)\right)}^{T}$,

$$\begin{array}{ll} \phi (O;\eta ,\theta ) & :=s(Z)\left[\frac{I(A=0)\tilde{q}(B|C,L)}{p(A|C,L)p(B|A,C,L)}\right.\\ & \;\{Y-m(A,B,C,L)\}+\left\{m(0,B^{g^{†}},C,L)-g(Z;\theta )\right\}] \end{array}$$

*and* $\mathbb{C}:=E_{P}\left[s(Z)▿g(Z;\theta )\right]$.

In practice, the variance‐covariance matrix in Theorem 4 above can be estimated using the following:

$$\begin{array}{l} {\hat{\mathbb{C}}}^{-1}{\mathbb{P}}_{n}\left\{s(Z)\left(\tilde{\phi }{\left(O;\hat{\eta },\hat{\theta }\right)}^{2}\right)s{\left(Z\right)}^{T}\right\}{\hat{\mathbb{C}}}^{{-1}^{T}} \end{array}$$

where $\hat{\mathbb{C}}={\mathbb{P}}_{n}\{s(Z)▿g(Z;\hat{\theta })\}$ and

$$\begin{array}{lll} \tilde{\phi }(O;\hat{\eta },\hat{\theta }) & :=Ŵ\{Y-\hat{m}(A,B,C,L)\} & \\ & \;+\left\{\hat{m}(0,B^{g^{†}},C,L)-g(Z;\hat{\theta })\right\} & \end{array}$$

For completeness, we also provide MSM outcome regression and inverse probability weighted estimators in Supporting Information S1: Appendix E.

## 6 Numerical Illustration: Simulation Studies

We conducted two different simulation studies. The first simulation study aims to compare the performance of the ORE, IPW, and TMLE estimators when the nuisance functions are estimated using parametric models under various model misspecification scenarios. The second simulation study aims to compare the performance of TMLE with ORE and IPW estimators when the nuisance functions are estimated through machine learning algorithms. In both simulation studies, we consider the proposed dietary substitution strategy indexed by (1) with x=max{supp(B)}.

### 6.1 Simulation Study 1: Illustrating Double Robustness of Proposed Estimator

Our first simulation study is designed to illustrate that our proposed estimator is more robust to model misspecification compared with estimators such as the IPW and ORE estimators. The simulation study was based on 1000 simulated data sets of sample sizes n=5000. We compared the bias, standard error, mean squared error, and 95% coverage probability of IPW, ORE, and TMLE estimators. We simulated the following variables: $\left(U,L,Ã,\tilde{B},\tilde{C},A,B,C,Y\right)$, where U∼Unif[0,1] denotes an unmeasured baseline covariate, L∼Ber(0.5) denotes a measured baseline covariate, ${\left(Ã,\tilde{B},\tilde{C}\right)}^{T}$ denotes a vector of variables that equals the vector of exposures ${\left(A,B,C\right)}^{T}$, where A∼Bin(3,expit(−2+L+U)), B∼Bin(3,expit(1−2L+U)), C∼Bin(3,expit(−1+L+U)), and Y∼Ber(expit(−1−A+B+C−2L)) denotes a binary outcome. We consider estimating the parameters in the following MSM given by $E(Y^{g}|L;\theta )=\text{expit}(\theta_{0}+\theta_{1}L)$.

The ORE estimator requires specifying a model for the outcome, the IPW estimator requires a model for the exposure, and the proposed TMLE requires both. To investigate robustness to misspecification of these models, we consider each of these estimators when: (i) all models are correctly specified (i.e., the data‐generating models were used), (ii) the outcome model is misspecified by assuming a working model for m(A,B,C,L) given by $\text{expit}(\beta_{0}+\beta_{1}A+\beta_{2}BL+\beta_{3}CL)$, and (iii) the exposure model is misspecified by assuming a working model for $\lambda_{b}(A,C,L)$ given by $\text{expit}(\alpha_{b0}+\alpha_{b1}A+\alpha_{b2}C)$, and the working model for $\lambda_{a}(C,L)$ given by $\text{expit}(\alpha_{a0}+\alpha_{a1}C)$.

**FIGURE 3:**
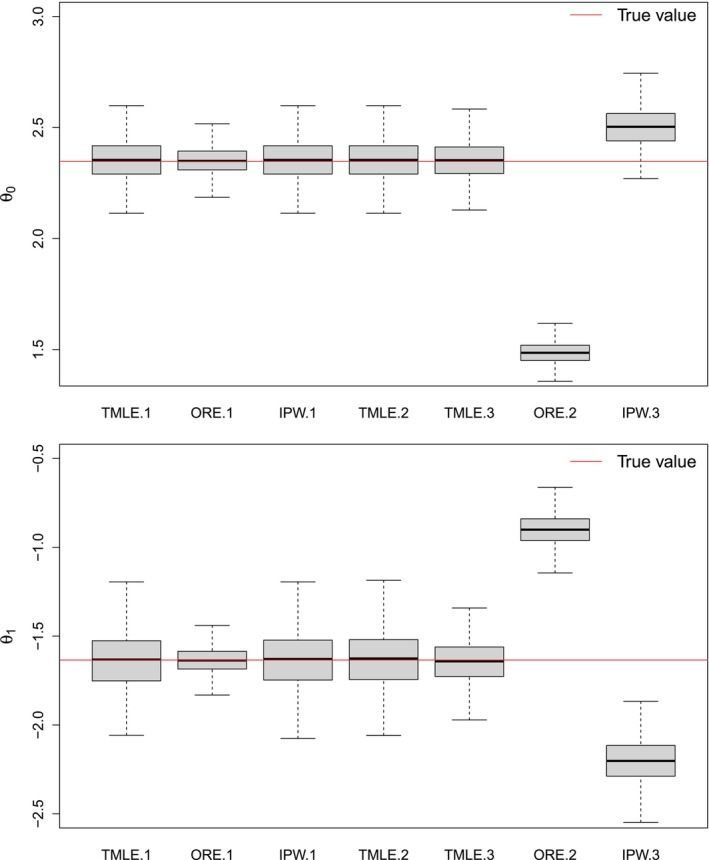
Findings from simulation study 1. *Top*: results for $\theta_{0}$. *Bottom*: results for $\theta_{1}$. “Method.1” denotes the method under the scenario where all models are correctly specified, “Method.2” denotes the method under the scenario where the outcome model is misspecified, and “Method.3” denotes the method under the scenario where the exposure model is misspecified. Each box plot shows the median, 25th–75th percentile and minimum‐maximum estimated parameter.

**FIGURE 4:**
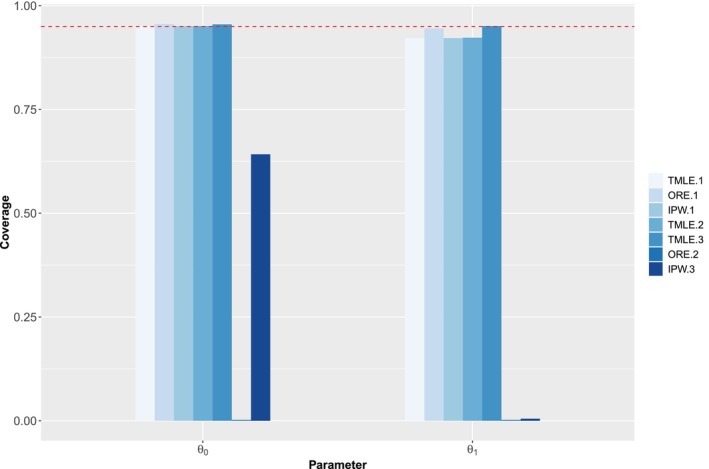
Coverage probability for simulation study 1. “Method.1” denotes the method under the scenario where all models are correctly specified, “Method.2” denotes the method under the scenario where outcome model is misspecified, and “Method.3” denotes the method under the scenario where the exposure model is misspecified.

Figures 3 and 4 (see also Table G.1 in Supporting Information S1: Appendix F) show the results from the simulation study. Consistent with our theoretical derivations, when all of the working models are correctly specified, all of the estimators are nearly unbiased. The TMLE estimator is also nearly unbiased in the two model misspecification settings whereas the IPW and ORE estimators are not all unbiased. As expected, ORE has the smallest standard error, and IPW and TMLE have comparable standard errors in all of the settings. With the exception of cases where working models for the nuisance functions are incorrectly specified, confidence intervals derived from the singly robust estimators typically achieve near nominal coverage. Our proposed doubly robust estimator, on the other hand, achieves near nominal coverage in all settings considered herein.

### 6.2 Simulation Study 2: Illustrating How to Incorporate Machine Learning Into Estimating Nuisance Functions

In the second simulation, we compare the performance of algorithms that use machine learning to estimate the nuisance functions for the parameters in an MSM. Specifically, we compare the proposed TMLE with IPW and ORE. Unlike the first simulation study, we add model complexity to the data‐generating mechanism by considering continuous covariates, which might mimic real‐life data more closely. We simulated 1000 hypothetical cohorts of n=(1000,5000,10000) comprising the following variables: $\left(U,L,Ã,\tilde{B},\tilde{C},A,B,C,Y\right)$, where U∼Unif[0,1] denotes an unmeasured baseline covariate; $L={\left(L_{1},L_{2}\right)}^{T}$ denotes measured baseline covariates where $L_{1}∼\text{Ber}(0.5)$ and $L_{2}∼\text{Unif}[0,2]$; ${\left(Ã,\tilde{B},\tilde{C}\right)}^{T}$ denotes a vector of variables that equals the vector of exposures ${\left(A,B,C\right)}^{T}$, where $A∼\mathrm{Bin}(3,\text{expit}(-2+0.75L_{1}+0.5L_{2}+U))$, $B∼\mathrm{Bin}(3,\text{expit}(-1+0.5L_{1}-0.25\exp(L_{2})+U))$, $C∼\mathrm{Bin}(3,\text{expit}(-1+L_{1}+U))$; and $Y∼\text{Ber}(\text{expit}(-1-A+B+C-2L_{1}+L_{2}-0.25\exp(L_{2})+0.25L_{1}L_{2}))$ denotes a binary outcome. We consider estimating the parameters in an MSM given by $g(Z;\theta )=\text{expit}(\theta_{0}+\theta_{1}L_{2})$.

Nuisance functions were estimated using the Highly Adaptive Lasso (HAL [35]) using the hal9001 R package [36]. The HAL is a non‐parametric regression function that can estimate infinite‐dimensional functional parameters by minimizing a loss‐specific empirical risk over a linear combination of indicator basis functions under the constraint that the sum of the absolute value of the coefficients is bounded by a constant [35, 37, 38]. Under the assumption that the nuisance functions have a finite sectional variation norm, HAL can estimate infinite‐dimensional functional parameters at an approximate rate of $n^{-1/3}$.

Table 1 compares the performance of the 3 estimators. The bias of ORE and IPW estimators is greater than those of TMLE in all scenarios. This is expected because the ORE and IPW estimators are not expected to converge at $\sqrt{n}$ rates when machine learning is used for nuisance parameter estimation, whereas TMLE allows the nuisance functions to converge at slower non‐parametric rates. Thus, the bias of TMLE tends to zero faster compared with the other estimators, albeit with a larger standard error compared with ORE. Moreover, the estimated coverage probability of the confidence intervals for TMLE based on the asymptotic variance gets closer to the nominal 95% as sample size increases. For instance, for ${\hat{\beta }}_{0}$, the 95% coverage probability is (91.2,93.2,94.2) for n=(1000,5000,10000), respectively. Additional simulation details and studies conducted using HAL, which illustrate similar results, are provided in Supporting Information S1: Appendix F.

**TABLE 1:** Results for simulation study for n=(1000,5000,10000) using non‐parametric models for nuisance functions: Bias, standard error (SE), and mean squared error (MSE). True (least false) value of $\left(\theta_{0},\theta_{1})=(0.933,0.268\right)$.

	n=1000			n=5000			n=10000		
ORE	Bias	SE	MSE	Bias	SE	MSE	Bias	SE	MSE
$\theta_{0}$	−0.124	0.192	0.196	−0.067	0.094	0.189	−0.049	0.068	0.194
$\theta_{1}$	0.042	0.180	0.223	0.042	0.092	0.204	0.038	0.068	0.202

IPW	Bias	SE	MSE	Bias	SE	MSE	Bias	SE	MSE
$\theta_{0}$	0.165	0.486	0.611	0.122	0.217	0.365	0.108	0.156	0.321
$\theta_{1}$	−0.279	0.518	0.735	−0.199	0.272	0.462	−0.179	0.202	0.418

TMLE	Bias	SE	MSE	Bias	SE	MSE	Bias	SE	MSE
$\theta_{0}$	−0.012	0.414	0.392	−0.014	0.182	0.244	−0.008	0.129	0.229
$\theta_{1}$	0.024	0.424	0.377	0.027	0.212	0.247	0.013	0.158	0.238

## 7 Application to the Nurses' Health Study

In the NHS, deaths are identified through a search of the vital statistics records of states and the National Death Index. Dietary assessments are collected every 4 years (e.g., 2010, 2014, and so forth) via a FFQ. In each FFQ, participants were asked how often, on average, they consumed each food item in standard portion sizes over the past year using nine multiple‐choice categories, ranging from “never or <1 time/month”, “1 per week”, “2–4 per week” to “≥6 times/day”. Given the sparse collection of dietary data and the fact that individuals were not asked about their planned dietary intake in the absence of intervention at baseline, we cannot emulate the target trial described in Section 2 without additional assumptions. To make progress, we assume that FFQ reported at baseline reflects the planned dietary intake that individuals would have reported at baseline.

To emulate the trial described in Section 2, we defined baseline as the time when participants returned the 2010 questionnaires. We identified individuals in the NHS who returned the 2010 questionnaire and satisfied all eligibility criteria, and excluded participants who reported implausible caloric intake (<500 or >3500 kcal/d) or had missing data on diet and covariates (about 0.9%). We considered the following two dietary strategies: $g_{1}$: *no intervention*; $g_{2}$: *replace all processed red meat with chicken, but do not exceed 7 servings of chicken per week (i.e*., x=7 *), while keeping other food groups at the intended levels in the absence of intervention*. The value x=7 corresponds to a recommendation of consuming at most 7 servings of chicken per week (or 1 serving of chicken per day). This value also aligns with current dietary guidelines in the United States, which recommends 26 ounces of meats (not including fish), poultry, or eggs on a weekly basis [39]. Eligible participants were followed from baseline (return of 2010 questionnaire) until death or 2 years after baseline, whichever occurred first. We applied the TMLE estimators to estimate the 2‐year cumulative risk under no intervention and the substitution intervention, as well as their risk difference. We modeled the nuisance functions using both parametric models and the HAL as described in the second simulation study. The models for the nuisance functions included as covariates the following baseline variables: age, body mass index, smoking status (never, past, current), exercise (hours/week), and aspirin intake (no use, 1–5 pills/week, ≥6 pills/week); baseline weekly intake for various food groups including processed red meat (included only in the outcome model and the model for chicken), chicken (included only in the outcome model), unprocessed red meat, fish, dairy, legumes, soy, nuts, fruits and vegetables, whole grains, sugar‐sweetened beverage, and alcohol; and past food intake for these various food groups measured in 2006.

**FIGURE 5:**
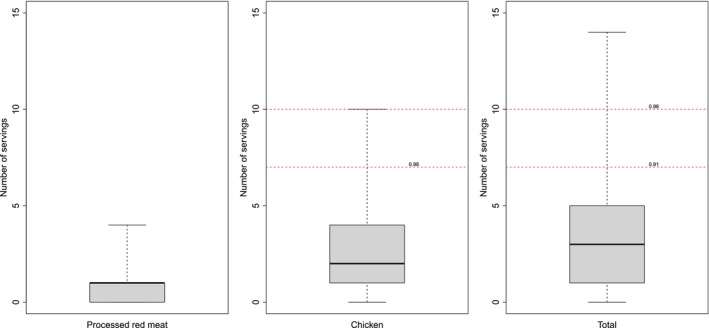
Boxplot of planned intake for processed red meat, chicken, and a total of processed red meat and chicken. Horizontal dashed lines indicate the range of values considered for the recommended maximum number of servings of chicken (x=7 to x=10) under the substitution intervention. Numbers on the dashed horizontal lines indicate the empirical cumulative distribution function evaluated at x=7 and x=10.

**FIGURE 6:**
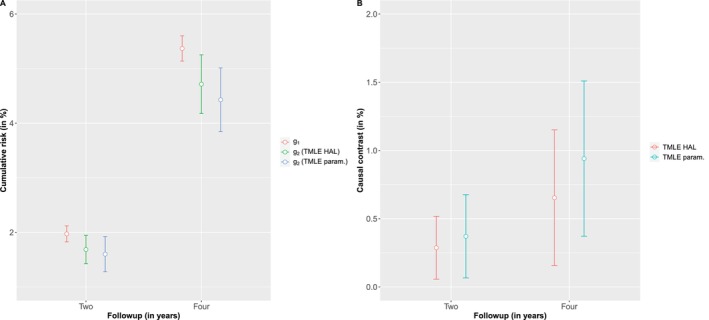
Estimated cumulative risk and causal contrast using TMLE with parametric models for estimating nuisance functions and TMLE with HAL for estimating nuisance functions.

A total of 33 621 NHS participants were identified as meeting the aforementioned inclusion criteria. The median (Q1, Q3) intake for processed red meat was 1 (Q1=0, Q3=2, respectively) serving per week, while for chicken it was 2 (Q1=1, Q3=4, respectively) servings per week (see Figure 5). Moreover, around 26% of study participants consumed no processed red meat, which aligns well with our substitution strategy. The estimated 2‐year mortality risk were 1.97% (95% CI: 1.82%–2.11%) under no intervention, and 1.68% (95% CI: 1.42%–1.94%) under the substitution intervention using TMLE with HAL. Therefore, based on TMLE with HAL, the risk difference under $g_{1}$ and $g_{2}$ was $\hat{\Delta }=0.29\%$ (95% CI: 0.06%–0.52%). That is, we estimated that the all‐cause mortality risk under the substitution strategy would be 0.29% lower compared with no intervention. Estimates from TMLE with parametric models were also similar (see Figure 6).

Sensitivity analysis was performed using various values of x={8,9,10} (see Supporting Information S1: Appendix G), and the results closely mirror those obtained when x=7. We also estimated the difference in the 4‐year cumulative risk under no intervention $g_{1}$ versus dietary substitution strategy $g_{2}$ over the 4‐year intervention period. The results, presented in Figure 6 and in Supporting Information S1: Appendix G, were similar to those from the 2‐year all‐cause mortality analysis.

## 8 Discussion

In this paper, we outlined a framework for estimating the average causal effect of dietary substitution strategies using observational data. We derived non‐parametric identification of these strategies and proposed new estimators that can be used in conjunction with parametric or non‐parametric estimation procedures. In contrast to existing research, our substitution strategies consider each individual's natural dietary intake and can be readily translated into real‐world interventions or recommendations. Moreover, compared with standard approaches, our estimators avoid imposing unrealistic assumptions of linearity and the absence of interaction terms in parametric outcome regression models.

Existing methods in literature often consider isocaloric substitution strategies by conditioning on total energy intake in parametric outcome models. This approach is problematic because it attempts to define a causal estimate based on an intervention targeting total energy intake rather than the specific foods themselves. Since total energy intake is a downstream variable of overall food intake, there are multiple ways to achieve the same level of total energy intake. Consequently, a strategy that fixes the total energy intake variable without specifying how individual foods should be modified to maintain isocaloric intake is generally considered an ill‐defined intervention. In practice, an isocaloric intervention can be implemented through a controlled feeding trial, wherein participants are provided with pre‐designed meals consisting of specified food items and precise quantities to meet macronutrient and caloric targets. However, conducting such an intervention in a long‐term trial is challenging. Therefore, we propose a strategy that approximates an isocaloric intervention while ensuring feasibility within the context of pragmatic trials. Specifically, we focus on substituting two food items with similar calorie content per serving while keeping other food components at baseline levels to ensure that outcome differences are primarily attributed to the substitution rather than caloric difference under the two strategies. In cases where the caloric content between the substitute and the food being replaced differs considerably, our method can be used to compare, for instance, replacing 1 unit of food 𝒜 with 1.5 units of food ℬ.

As shown in Supporting Information S1: Appendix A, implementing our substitution strategies in a real randomized controlled trial requires collecting (a) the planned/intended diet at the beginning of the study, which provides the information to guide participants on how to modify their diets, and (b) actual intake during the study period, which provide the information to assess adherence. Without information on the planned intake, we cannot differentiate whether participants adhere to or deviate from the protocol. For example, if a participant ate 0 servings of processed red meat, 1 serving of chicken, and 2 servings of fish per week under the dietary substitution intervention, it is unclear whether the participant replaced processed red meat with chicken or fish. Since the effect estimates depend on the food that is used as a replacement, collecting the planned diet intake would be important for estimating the effect of the specific diet substitution strategy. In this study, due to the absence of data on planned dietary intake, we rely on a strong determinism assumption. When data on planned intake is available, this determinism is not needed and the identification formula remains virtually the same. The only adjustment is that the distribution of actual intake A, B, and C in the identifying formula is replaced with that of planned intake Ã, $\tilde{B}$, and $\tilde{C}$, respectively.

In lieu of the determinism assumption, researchers may consider two alternative estimands to the one proposed here. The identifying formulas for these alternatives would coincide with the formula provided herein under certain conditions. First, one could instead assume that the distribution of planned intake in the study population is identical to that of actual intake in the same population. This allows for an intervention in which, for example, chicken intake is assigned based on a random draw from the distributions of planned processed red meat and planned chicken intake in the study population. However, this approach remains unsatisfactory in the dietary contexts, as participants may be assigned to consume more or less than what they typically eat. Alternatively, researchers may choose to consider a dietary intervention that modifies diet based on actual food intake, analogous to Haneuse and Rotnitzky [9]. Under the weaker assumptions (i.e., Assumptions 1, 3, and 4), our estimated risk difference can be interpreted as the difference in mortality risk if individuals in our study population, contrary to the fact, had substituted the processed red meat they consumed with equivalent servings of chickens, compared to the mortality risk under no intervention. This causal contrast represents an intervention that cannot be feasibly implemented in real‐world settings, as it is impossible to retrospectively change an individual's intake. In our paper, we have chosen to focus on causal inference that is clearly defined by variables representing interventions that are, at least in principle, implementable. Our approach aligns with the longstanding advocacy for an “interventionist” perspective in causal analyses within statistics [10, 40, 41].

## Conflicts of Interest

The authors declare no conflicts of interest.

## Supporting information




**Data S1**.

## Data Availability

The data that support the findings of this study are available from Nurses' Health Studies. Restrictions apply to the availability of these data, which were used under license for this study. Data are available from https://www.nurseshealthstudy.org/researchers with the permission of Nurses' Health Studies.
