# Supplementary material for: Identification and Estimation of the Average Causal Effects Under Dietary Substitution Strategies
Source: Stat Med. 2025 Feb 20;44(5):e70007. doi: 10.1002/sim.70007 (PMC11840885; doi:10.1002/sim.70007)
Supplement: Supplementary file 1 — Data S1. [file SIM-44-0-s001.pdf]

WEB APPENDIX FOR "IDENTIFICATION AND ESTIMATION OF CAUSAL EFFECTS UNDER FOOD SUBSTITUTION STRATEGIES"

A. Observed data and dietary substitution strategies

A.1 Observed data Figure G.1 shows a more realistic Directed Acyclic Graph (DAG) that captures the complexities of a one-week observational data structure. In this DAG, we let $k{=}0,...,K$ denote arbitrary follow-up intervals with interval $k{=}K$ corresponding to the final time interval in a week. In addition to the notations introduced in the main text, we let (A_k,B_k,C_k) denote the intake of $\mathcal{A}$, $\mathcal{B}$ and $\mathcal{C}$ in time interval k, respectively, and let U_k denote underlying factors that affect (A_k,B_k,C_k) (for $k{=}0,...,K$).

This DAG does not capture the deterministic relationship between planned weekly intake and actual weekly intake that we have assumed in the main text. However, we can still assume that no matter how people eat their food during the week, how much they plan to eat (e.g., based on their weekly groceries) and how much they actually eat will approximately be equal for the entire week. Thus, under the assumptions described in main text, our identification strategy of the average causal effect of dietary substitution strategies remains valid for the DAG in Figure G.1.

[Figure 1 about here.]

A.2 Dietary substitution strategies Here we show that different substitution strategies can have different impacts on clinical outcomes. Building upon our motivating example, consider treatment strategy g defined as:

- g: swap the portions of $\mathcal{A}$ and $\mathcal{B}$, but keep $\mathcal{C}$ at a baseline level in the absence of intervention.

In isocaloric intervention of food substitution (i.e., substitution of portions between two food groups $\mathcal{A}$ and $\mathcal{B}$ that are similar to one another in calories; e.g., replacing 1 portion of steak for 1 portion of chicken), g is feasible as it is reasonable to assume that swapping food groups that are similar in calories will not result in changes in intake of other diet groups (group $\mathcal{C}$), and as such g is a substitution recommendation that is sustainable in the long run. On the other hand, if the substitution of portions between two food groups $\mathcal{A}$ and $\mathcal{B}$ is drastically different in calories (e.g., replacing 1 portion of steak for 1 portion vegetables), then g will result in a hypocaloric diet, which

is challenging to sustain over a long period of time and therefore not as practically relevant. In this case, an alternative strategy that allows people to choose their diet intake after taking into consideration the substitution strategy of $\mathcal{A}$ and $\mathcal{B}$ is more feasible, as it is more reasonable to assume that most people would want to change their food intake in other diet groups ($\mathcal{C}$) to make up for any losses in caloric intake. An example of such a strategy is given by g':

- g': swap the portions of $\mathcal{A}$ and $\mathcal{B}$ but *subsequently* allow the participant to modify their portions for $\mathcal{C}$.

A.2.1 A closer look at treatment strategy g': identification requires multiple time points. From the outset, g' appears to be feasible for the second scenario where the substitution of portions between two food groups $\mathcal{A}$ and $\mathcal{B}$ is drastically different in calories. However, the identification of a causal estimand under g', i.e., $E(Y^{g'})$, can be more challenging in practice. To see this, we first note that g' inherently implies a temporal sequence of events, as the portions of food group $\mathcal{C}$ under intervention depends on the portions of food groups $\mathcal{A}$ and $\mathcal{B}$ under the intervention. As such, in order for $E(Y^{g'})$ to be identified using observed data, g' requires at least two consecutive measurements that can capture the decisions that one makes regarding their food intake portions on $\mathcal{A}$, $\mathcal{B}$ and $\mathcal{C}$ over time.

A.3 A hypothetical randomized controlled trial Here we illustrate the timeline for a hypothetical randomized controlled trial of the dietary substitution intervention described in the main paper. The implementation of the dietary substitution intervention may proceed as follows in an actual randomized controlled trial: at the beginning of each week, participants would be asked about the weekly servings of each food they anticipate consuming if their diet was not intervened upon. If an individual intended to consume, for example, two servings of processed red meat and one serving of chicken (hereafter referred to as the *intended intake*), then they would be instructed to eat three servings of chicken and zero processed red meat. To assess adherence to the assigned dietary strategy, participants would also be asked about what they actually ate (hereafter referred to as the *actual intake*) in the previous week.

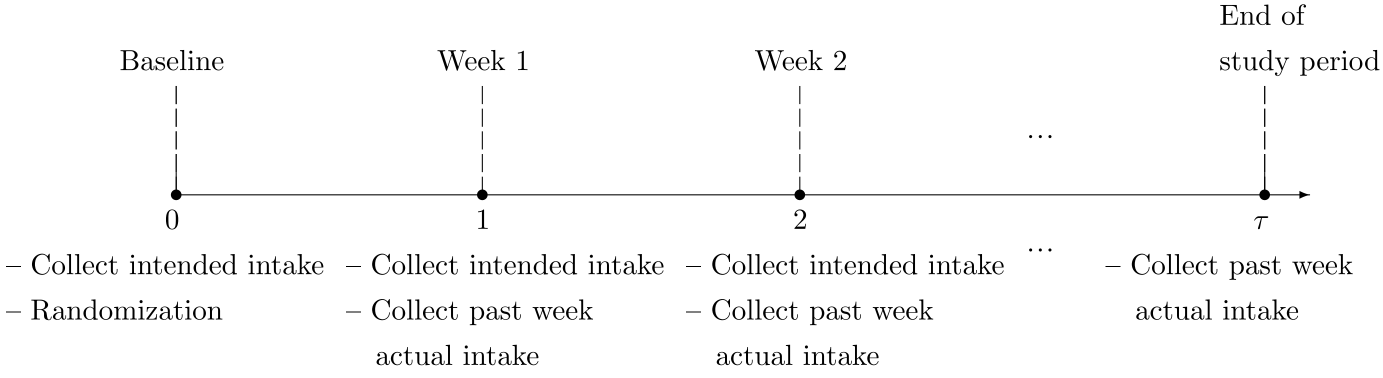

B. Identification proofs

B.1 Proof of Theorem 1 Here we prove that the average counterfactual outcome under the food substitution policy given in the main manuscript can be identified using the formula given in Theorem 1 of the main manuscript.

Theorem 1 states that under Assumptions 1–4, the counterfactual outcome under the food substitution policy g (defined in the main manuscript) is given by:

$$\mu^g = \sum_{a,b,c,l} E(Y \mid A{=}0, B{=}d(a,b), C{=}c, L{=}l) p(a,b,c \mid l) p(l) \tag{B.1}$$

Proof. We first note that under point exposure settings, a constant dietary behaviour is assumed throughout the study period in the observational study (i.e., intended and actual intake remains fairly consistent each week from the start of the study). As such, to obtain point identification, we borrow information from those in the observation study whose actual dietary intake matches possible intervention dietary intake levels, and assume their dietary behavior stayed constant throughout the study period. Another way to view this is that had actual weekly dietary intake at baseline, contrary to fact, taken a value at the intervention intake levels, (e.g., see $A^{g^\dagger}$, $B^{g^\dagger}$ and $C^{g^\dagger}$ in the SWIG in Figure 2 of main text), then subsequent dietary intake levels would remain at intervention intake levels until the end of the study period.

$$\begin{aligned}
E(Y^g) &= \sum_{\tilde{a},\tilde{b},\tilde{c},l} E(Y^g \mid \tilde{A}{=}\tilde{a}, \tilde{B}{=}\tilde{b}, \tilde{C}{=}\tilde{c}, L{=}l) p(\tilde{a},\tilde{b},\tilde{c} \mid l) p(l) \\
&= \sum_{\tilde{a},\tilde{b},\tilde{c},l} E(Y^g \mid \tilde{A}{=}\tilde{a}, \tilde{B}{=}\tilde{b}, \tilde{C}{=}\tilde{c}, B^{g^\dagger}{=}d(\tilde{a},\tilde{b}), L{=}l) p(\tilde{a},\tilde{b},\tilde{c} \mid l) p(l) && \text{(B.2)} \\
&= \sum_{\tilde{a},\tilde{b},\tilde{c},l,b^\dagger} E(Y^g \mid \tilde{A}{=}\tilde{a}, \tilde{B}{=}\tilde{b}, \tilde{C}{=}\tilde{c}, B^{g^\dagger}{=}b^\dagger, L{=}l) I(b^\dagger{=}d(\tilde{a},\tilde{b})) p(\tilde{a},\tilde{b},\tilde{c} \mid l) p(l) \\
&= \sum_{\tilde{a},\tilde{b},\tilde{c},l,b^\dagger} E(Y^{a=0,b=b^\dagger} \mid \tilde{A}{=}\tilde{a}, \tilde{B}{=}\tilde{b}, \tilde{C}{=}\tilde{c}, B^{g^\dagger}{=}b^\dagger, L{=}l) I(b^\dagger{=}d(\tilde{a},\tilde{b})) p(\tilde{a},\tilde{b},\tilde{c} \mid l) p(l) \\
& && \text{(B.3)} \\
&= \sum_{\tilde{a},\tilde{b},\tilde{c},l,b^\dagger} E(Y^{a=0,b=b^\dagger} \mid \tilde{A}{=}\tilde{a}, \tilde{B}{=}\tilde{b}, \tilde{C}{=}\tilde{c}, L{=}l) I(b^\dagger{=}d(\tilde{a},\tilde{b})) p(\tilde{a},\tilde{b},\tilde{c} \mid l) p(l) && \text{(B.4)} \\
&= \sum_{\tilde{a},\tilde{b},\tilde{c},l,b^\dagger} E(Y^{a=0,b=b^\dagger} \mid A{=}\tilde{a}, B{=}\tilde{b}, C{=}\tilde{c}, L{=}l) I(b^\dagger{=}d(\tilde{a},\tilde{b})) p(\tilde{a},\tilde{b},\tilde{c} \mid l) p(l) && \text{(B.5)}
\end{aligned}$$

$$= \sum_{\tilde{a},\tilde{b},\tilde{c},l,b^\dagger} E(Y^{a=0,b=b^\dagger}|A=0,B=b^\dagger,C=\tilde{c},L=l)I(b^\dagger=d(\tilde{a},\tilde{b}))p(\tilde{a},\tilde{b},\tilde{c}|l)p(l) \quad \text{(B.6)}$$

$$= \sum_{\tilde{a},\tilde{b},\tilde{c},l,b^\dagger} E(Y|A=0,B=b^\dagger,C=\tilde{c},L=l)I(b^\dagger=d(\tilde{a},\tilde{b}))p(\tilde{a},\tilde{b},\tilde{c}|l)p(l) \quad \text{(B.7)}$$

$$= \sum_{\tilde{a},\tilde{b},\tilde{c},l} E(Y|A=0,B=d(\tilde{a},\tilde{b}),C=\tilde{c},L=l)p(\tilde{a},\tilde{b},\tilde{c}|l)p(l)$$

$$= \sum_{a,b,c,l} E(Y|A=0,B=d(a,b),C=c,L=l)p(a,b,c|l)p(l) \quad \text{(B.8)}$$

In addition to probability rules, we note the following conditions that were used in the proof. Lines (B.2) and (B.4) follow from the fact that $B^{g^\dagger}=d(\tilde{A},\tilde{B})$ is defined as a deterministic function of intended variables $\tilde{A}$ and $\tilde{B}$. Line (B.3) follows from the fact that when $B^{g^\dagger}=b^\dagger$ and $\tilde{C}=C^{g^\dagger}=\tilde{c}$, then $C=\tilde{c}$ and $Y^g=Y^{a=0,b=b^\dagger,c=\tilde{c}}=Y^{a=0,b=b^\dagger}$. Finally, line (B.5) follows by determinism (since conditioning on $\{\tilde{A}=\tilde{a},\tilde{B}=\tilde{b},\tilde{C}=\tilde{c},L=l\}$ is the same as conditioning on $\{A=\tilde{a},B=\tilde{b},C=\tilde{c},L=l\}$), line (B.6) follows by exchangeability, line (B.7) follows by consistency, and line (B.8) follows by rewriting the indices. □

We can also derive the inverse probability weighted representation of the identifying formula in the following manner:

$$E(Y^g)=\sum_{a,b,c,l} E(Y|A=0,B=d(a,b),C=c,L=l)p(a,b,c|l)p(l)$$

$$= \sum_{a,b,c,l,b^\dagger} E(Y|A=0,B=b^\dagger,C=c,L=l)\underbrace{I(b^\dagger=d(a,b))}_{q(b^\dagger|a,b,c,l)}p(a,b,c|l)p(l)$$

$$= \sum_{c,l,b^\dagger} E(Y|A=0,B=b^\dagger,C=c,L=l)\underbrace{\left\{\sum_{a,b} q(b^\dagger|a,b,c,l)p(a,b|c,l)\right\}}_{\tilde{q}(b^\dagger|c,l)}p(c|l)p(l)$$

$$= \sum_{a,c,b^\dagger,l} E(Y|A=a,B=b^\dagger,C=c,L=l)\frac{I(a=0)\tilde{q}(b^\dagger|c,l)}{p(a|b^\dagger,c,l)p(b^\dagger|c,l)}p(a|b^\dagger,c,l)p(b^\dagger|c,l)p(c|l)p(l)$$

$$= E\left[Y\frac{I(A=0)\tilde{q}(B|C,L)}{P(A|B,C,L)P(B|C,L)}\right] = E\left[Y\frac{I(A=0)\tilde{q}(B|C,L)}{P(A|C,L)P(B|A,C,L)}\right]$$

where $p(\cdot)$ ($p(\cdot|\cdot)$) denote (conditional) probability distribution of the observed data, $q(b^\dagger|a,b)$ denotes the deterministic distribution of $B^{g^\dagger}$ as defined by the food substitution policy, and $\tilde{q}(b^\dagger|c,l)$ denotes an implied distribution of $B^{g^\dagger}$ after marginalizing over A and B.

B.2 Derivation of $\tilde{q}(B|A,L)$ for the substitution strategy considered in the main manuscript We now derive the expression for $\tilde{q}(B|A,L)$ for the following substitution strategy: If $x{=}\max(\text{supp}(B))$, we define $d(a,b)$ (with some slight abuse in notation where $\tilde{A}{=}a$, and $\tilde{B}{=}b$) in (B.2) as follows:

$$d(a,b)=\begin{cases} a+b, & \text{if } a+b\leq x \\ x, & \text{if } a+b>x \end{cases}$$

In this case,

$$q(b^\dagger|a,b,c,l)=I(b^\dagger=a+b)I(a+b\leq x)+I(b^\dagger=x)I(a+b>x)$$

and thus we have the following derivation for the expression of $\tilde{q}(b^\dagger|c,l)$:

$$\begin{aligned}
\tilde{q}(b^\dagger|c,l) &= \sum_{a\geq 0,b\geq 0} q(b^\dagger|a,b,c,l)p(a,b|c,l) \\
&= \sum_{a\geq 0,b\geq 0} \big\{I(b^\dagger=a+b)I(a+b\leq x)+I(b^\dagger=x)I(a+b>x)\big\}p(a,b|c,l) \\
&= \sum_{a\geq 0,b\geq 0} I(b=b^\dagger-a)I(b^\dagger\leq x)p(a,b|c,l)+\sum_{a\geq 0,b\geq 0} I(b^\dagger=x)I(a+b>x)p(a,b|c,l) \\
&= \sum_{0\leq a\leq b^\dagger} I(b^\dagger\leq x)p_B(b^\dagger-a|a,c,l)p(a|c,l)+\sum_{a\geq 0,b\geq 0} I(b^\dagger=x)I(a+b>x)p(a,b|c,l) \\
&= \sum_{a\geq 0} I(a\leq b^\dagger\leq x)p_B(b^\dagger-a|a,c,l)p(a|c,l)+\sum_{a\geq 0,b\geq 0} I(b^\dagger=x)I(b>x-a)p(a,b|c,l) \\
&= \sum_{a\geq 0}\Bigg\{I(a\leq b^\dagger\leq x)p_B(b^\dagger-a|a,c,l)+I(b^\dagger=x)[1-F_B(x-a|a,c,l)]\Bigg\}p(a|c,l)
\end{aligned}$$

where the summation in the fourth equality holds because of the substitution given by $b{=}b^\dagger-a$. By this substitution step, $b\geq 0$ implies that $b^\dagger-a\geq 0$, and so it must be that $a\leq b^\dagger$. Intuitively, it must be the case that only those whose $b^\dagger-a\geq 0$ will contribute to the summation and thus it must be that $a\leq b^\dagger$.

B.3 An alternative substitution strategy We may also consider the following strategy that does not to force all individuals to target $A^{g^\dagger}{=}0$. This might be of more interest in e.g., scenarios where median(A)>>median(B) (i.e., in cases where people tend to eat much more of $\mathcal{A}$ than $\mathcal{B}$):

$$d_B(a,b)=\begin{cases} a+b, & \text{if } a+b\leq x \\ x, & \text{if } a+b>x \end{cases} \tag{B.9}$$

$$d_A(a,b) = \begin{cases} 0, & \text{if } a+b \leq x \\ a+b-x, & \text{if } a+b > x \end{cases} \tag{B.10}$$

where $x = \max(\text{supp}(B))$. To identify the average counterfactual outcome under the above strategy, we posit the following set of assumptions:

Assumption B.1 (Consistency). *If $A=a$, $B=b$ and $C=c$, then $Y^{a,b,c}=Y$.*

Assumption B.2 (Determinism). $\tilde{A}=A$, $\tilde{B}=B$, $\tilde{C}=C$ (with probability 1)

Assumption B.3 (Positivity). *If $(a,b,c,l) \in supp(A^{g^\dagger}, B^{g^\dagger}, C, L)$, then $(a,b,c,l) \in supp(A,B,C,L)$.*

Assumption B.4 (Exchangeability). $Y^{a,b} \perp\!\!\!\perp (A,B) | C, L$, $\forall b \in supp(B^{g^\dagger})$, $\forall a \in supp(A^{g^\dagger})$

Theorem B.1. *Under Assumptions B.1–B.4, the counterfactual outcome under the food substitution policy g is given by:*

$$\mu^g = \sum_{a,b,c,l} E(Y|A=d_A(a,b), B=d_B(a,b), C=c, L=l)p(a,b,c|l)p(l) \tag{B.11}$$

Proof.

$$E(Y^g) = \sum_{\tilde{a},\tilde{b},\tilde{c},l} E(Y^g|\tilde{A}=\tilde{a}, \tilde{B}=\tilde{b}, \tilde{C}=\tilde{c}, L=l)p(\tilde{a},\tilde{b},\tilde{c}|l)p(l)$$

$$= \sum_{\tilde{a},\tilde{b},\tilde{c},l} E(Y^g|\tilde{A}=\tilde{a}, \tilde{B}=\tilde{b}, \tilde{C}=\tilde{c}, A^{g^\dagger}=d_A(\tilde{a},\tilde{b}), B^{g^\dagger}=d_B(\tilde{a},\tilde{b}), L=l)p(\tilde{a},\tilde{b},\tilde{c}|l)p(l) \tag{B.12}$$

$$= \sum_{\tilde{a},\tilde{b},\tilde{c},l,b^\dagger} E(Y^g|\tilde{A}=\tilde{a}, \tilde{B}=\tilde{b}, \tilde{C}=\tilde{c}, B^{g^\dagger}=b^\dagger, L=l)I(b^\dagger=d_B(\tilde{a},\tilde{b}))p(\tilde{a},\tilde{b},\tilde{c}|l)p(l)$$

$$= \sum_{\tilde{a},\tilde{b},\tilde{c},l,a^\dagger,b^\dagger} E(Y^{a=a^\dagger,b=b^\dagger}|\tilde{A}=\tilde{a}, \tilde{B}=\tilde{b}, \tilde{C}=\tilde{c}, A^{g^\dagger}=a^\dagger, B^{g^\dagger}=b^\dagger, L=l) \tag{B.13}$$
$$I[(a^\dagger,b^\dagger)=(d_A(\tilde{a},\tilde{b}), d_B(\tilde{a},\tilde{b}))]p(\tilde{a},\tilde{b},\tilde{c}|l)p(l)$$

$$= \sum_{\tilde{a},\tilde{b},\tilde{c},l,a^\dagger,b^\dagger} E(Y^{a=a^\dagger,b=b^\dagger}|\tilde{A}=\tilde{a}, \tilde{B}=\tilde{b}, \tilde{C}=\tilde{c}, L=l) \tag{B.14}$$
$$I[(a^\dagger,b^\dagger)=(d_A(\tilde{a},\tilde{b}), d_B(\tilde{a},\tilde{b}))]p(\tilde{a},\tilde{b},\tilde{c}|l)p(l)$$

$$= \sum_{\tilde{a},\tilde{b},\tilde{c},l,a^\dagger,b^\dagger} E(Y^{a=a^\dagger,b=b^\dagger}|A=\tilde{a}, B=\tilde{b}, C=\tilde{c}, L=l) \tag{B.15}$$

$$I[(a^\dagger,b^\dagger)=(d_A(\tilde{a},\tilde{b}),d_B(\tilde{a},\tilde{b}))]p(\tilde{a},\tilde{b},\tilde{c}|l)p(l)$$

$$=\sum_{\tilde{a},\tilde{b},\tilde{c},l,a^\dagger,b^\dagger} E(Y^{a=a^\dagger,b=b^\dagger}|A=0,B=b^\dagger,C=\tilde{c},L=l)I[(a^\dagger,b^\dagger)=(d_A(\tilde{a},\tilde{b}),d_B(\tilde{a},\tilde{b}))]p(\tilde{a},\tilde{b},\tilde{c}|l)p(l) \tag{B.16}$$

$$=\sum_{\tilde{a},\tilde{b},\tilde{c},l,a^\dagger,b^\dagger} E(Y|A=a^\dagger,B=b^\dagger,C=\tilde{c},L=l)I[(a^\dagger,b^\dagger)=(d_A(\tilde{a},\tilde{b}),d_B(\tilde{a},\tilde{b}))]p(\tilde{a},\tilde{b},\tilde{c}|l)p(l) \tag{B.17}$$

$$=\sum_{\tilde{a},\tilde{b},\tilde{c},l} E(Y|A=d_A(\tilde{a},\tilde{b}),B=d_B(\tilde{a},\tilde{b}),C=\tilde{c},L=l)p(\tilde{a},\tilde{b},\tilde{c}|l)p(l)$$

$$=\sum_{a,b,c,l} E(Y|A=d_A(a,b),B=d_B(a,b),C=c,L=l)p(a,b,c|l)p(l) \tag{B.18}$$

In addition to probability rules, we note the following conditions that were used in the proof. Lines (B.12) and (B.14) follow from the fact that $A^{g^\dagger}=d_A(\tilde{A},\tilde{B})$ and $B^{g^\dagger}=d_B(\tilde{A},\tilde{B})$ are defined as a deterministic function of $\tilde{A}$ and $\tilde{B}$. Line (B.13) follows from the fact that when $A^{g^\dagger}=a^\dagger$, $B^{g^\dagger}=b^\dagger$ and $\tilde{C}=C^{g^\dagger}=\tilde{c}$, then $C=\tilde{c}$ and $Y^g=Y^{a=a^\dagger,b=b^\dagger,c=\tilde{c}}=Y^{a=a^\dagger,b=b^\dagger}$. Finally, line (B.15) follows by determinism, line (B.16) follows by exchangeability, line (B.17) follows by consistency, and line (B.18) follows by rewriting indices. □

We can also derive the inverse probability weighted representation of the identifying formula in the following manner:

$$E(Y^g)=\sum_{a,b,c,l} E(Y|A=d_A(a,b),B=d_B(a,b),C=c,L=l)p(a,b,c|l)p(l)$$

$$=\sum_{a,b,c,l,a^\dagger,b^\dagger} E(Y|A=a^\dagger,B=b^\dagger,C=c,L=l)\underbrace{I[(a^\dagger,b^\dagger=(d_A(a,b),d_B(a,b))]}_{q(a^\dagger,b^\dagger|a,b,c,l)}p(a,b,c|l)p(l)$$

$$=\sum_{c,l,a^\dagger,b^\dagger} E(Y|A=a^\dagger,B=b^\dagger,C=c,L=l)\underbrace{\left\{\sum_{a,b} q(a^\dagger,b^\dagger|a,b,c,l)p(a,b|c,l)\right\}}_{\tilde{q}(a^\dagger,b^\dagger|c,l)}p(c|l)p(l)$$

$$=\sum_{c,l,a^\dagger,b^\dagger} E(Y|A=a^\dagger,B=b^\dagger,C=c,L=l)\frac{\tilde{q}(a^\dagger,b^\dagger|c,l)}{p(a^\dagger|b^\dagger,c,l)p(b^\dagger|c,l)}p(a^\dagger|b^\dagger,c,l)p(b^\dagger|c,l)p(c|l)p(l)$$

$$=E\left[Y\frac{\tilde{q}(A,B|C,L)}{P(A,B|C,L)}\right]$$

We now derive the expression for $\tilde{q}(A,B|C,L)$ for the following substitution strategy g defined by (B.9) and (B.10).

$$\begin{aligned}
\tilde{q}(a^\dagger,b^\dagger|c,l) &= \sum_{a\geq 0,b\geq 0} q(a^\dagger,b^\dagger|a,b,c,l)p(a,b|c,l) \\
&= \sum_{a\geq 0,b\geq 0} \big\{I[(a^\dagger,b^\dagger)=(0,a+b)]I(a+b\leq x)+I[(a^\dagger,b^\dagger)=(a+b-x,x))]I(a+b>x)\big\}p(a,b|c,l) \\
&= \sum_{a\geq 0,b\geq 0} I(a^\dagger=0)I(b=b^\dagger-a)I(b^\dagger\leq x)p(a,b|c,l)+ \\
&\quad \sum_{a\geq 0,b\geq 0} I(a=a^\dagger-b+x)I(b^\dagger=x)I(a+b>x)p(a,b|c,l) \\
&= \sum_{0\leq a} I(a^\dagger=0)I(a\leq b^\dagger\leq x)p_B(b^\dagger-a|a,c,l)p(a|c,l)+ \\
&\quad \sum_{0\leq b<a^\dagger+x} I(a^\dagger>0)I(b^\dagger=x)p_A(a^\dagger-b+x|b,c,l)p(b|c,l)
\end{aligned}$$

Thus, we have the following inverse probability weighted representation of the identifying formula for this particular g:

$$\begin{aligned}
&E\left[Y\left\{\frac{\tilde{q}(A,B|C,L)}{P(A,B|C,L)}\right\}\right]= \\
&E\left[Y\left\{\frac{\sum_{a\geq 0}I(A=0)I(a\leq B\leq x)p_B(B-a|a,C,L)p(a|C,L)}{P(A,B|C,L)}+\right.\right. \\
&\qquad\left.\left.\frac{\sum_{b\geq 0}I(A>b-x,A>0)I(B=x)p_A(A-b+x|b,C,L)p(b|C,L)}{P(A,B|C,L)}\right\}\right]
\end{aligned}$$

C. Derivation of efficient influence function

C.1 Derivation of the efficient influence function for μ^g

Proof. The efficient influence function in the nonparametric model $\mathcal{M}_{np}$ is defined as the unique mean zero, finite variance random variable $\varphi^{\text{eff}}(O)$ such that

$$\frac{d\mu^g(P_\epsilon)}{d\epsilon}\Big|_{\epsilon=0} = E\{\varphi^{\text{eff}}(O)S(O)\}$$

where $O=(L,A,B,C,Y)$, $d\mu^g(P_\epsilon)/d\epsilon|_{\epsilon=0}$ is known as the pathwise derivative of parameter μ^g along a parametric submodel indexed by ϵ, and $S(O)$ is the score function of the parametric submodel evaluated at $\epsilon=0$ (such that $P_{\epsilon=0}=P$). The efficient influence function of μ^g can be realized by using differentiation rules:

$$\begin{aligned}
\frac{d\mu^g(P_\epsilon)}{d\epsilon}\Big|_{\epsilon=0} &= \frac{d}{d\epsilon}E_P\Big\{E_{P_\epsilon}(Y|A=0,B^{g^\dagger},C,L)\Big\}\Big|_{\epsilon=0} + \frac{d}{d\epsilon}E_{P_\epsilon}\Big\{E_P(Y|A=0,B^{g^\dagger},C,L)\Big\}\Big|_{\epsilon=0} \\
&= \frac{d}{d\epsilon}E_P\Big\{E_{P_\epsilon}(Y|A=0,B^{g^\dagger},C,L)\Big\}\Big|_{\epsilon=0} + \frac{d}{d\epsilon}E_{P_\epsilon}\Big\{m(0,B^{g^\dagger},C,L)\Big\}\Big|_{\epsilon=0} \\
&= E_P\Big[E_P\Big\{YS(Y|A=0,B^{g^\dagger},C,L)|A=0,B^{g^\dagger},C,L\Big\}\Big] + \\
&\quad E_P\Big\{m(0,B^{g^\dagger},C,L)S(A,B,C,L)\Big\} \\
&= E_P\Big\{\frac{I(A=0)\tilde{q}(B|C,L)}{p(A,B|C,L)}YS(Y|A,B,C,L)\Big\} + \\
&\quad E_P\Big[\Big\{m(0,B^{g^\dagger},C,L)-\mu^g\Big\}S(A,B,C,L)\Big] \\
&= E_P\Big\{\frac{I(A=0)\tilde{q}(B|C,L)}{p(A,B|C,L)}(Y-m(A,B,C,L))S(Y|A,B,C,L)\Big\} + \\
&\quad E_P\Big[\Big\{m(0,B^{g^\dagger},C,L)-\mu^g\Big\}S(O)\Big] \\
&= E_P\Big\{\frac{I(A=0)\tilde{q}(B|C,L)}{p(A,B|C,L)}(Y-m(A,B,C,L))S(O)\Big\} + \\
&\quad E_P\Big[\Big\{m(0,B^{g^\dagger},C,L)-\mu^g\Big\}S(O)\Big]
\end{aligned}$$

Thus, the efficient influence function for μ^g is given by:

$$\varphi^{\text{eff}}(O) = \frac{I(A=0)\tilde{q}(B|C,L)}{p(A,B|C,L)}(Y-m(A,B,C,L)) + \Big\{m(0,B^{g^\dagger},C,L)-\mu^g\Big\},$$

where

$$\tilde{q}(B|C,L) := \Big[\sum_a \{I(a\leq B\leq x)P(B-a|a,C,L) + I(B=x)[1-F_B(x-a|a,C,L)]\}p_A(a|c,L)\Big].$$

□

C.2 Derivation of the efficient influence function for parameters in the MSM Following Petersen et al. (2014) (see also Zheng et al., 2016), our causal quantity of interest for a binary outcome Y is defined as:

$$\theta(P)=\underset{\theta\in\mathbf{R}^p}{\operatorname{argmin}}\ E\Big[\xi(L;P)\log\{g(Z;\theta)\}+\{1-\xi(L;P)\}\log\{1-g(Z;\theta)\}\Big].$$

where $\xi(L;P)=E_P\Big\{m(0,B^{g^\dagger},C,L)|L\Big\}=E(Y^g|L)$, $g(Z;\theta):=\text{expit}\{s(Z)^T\theta\}$ where $s(Z)$ is a vector of linear predictors with the same dimensions as θ. Such a vector of $\theta(P)$ solves the equation:

$$0=E_P\left[\frac{\nabla g(Z;\theta(P))}{g(Z;\theta(P))(1-g(Z;\theta(P)))}\{\xi(L;P)-g(Z;\theta(P))\}\right], \tag{C.19}$$

where $\nabla g(Z;\theta)=\frac{dg(Z;\theta)}{d\theta}$. Under our setting, can be further reduced to:

$$0=E_P[s(Z)\{\xi(L;P)-g(Z;\theta(P))\}].$$

Our aim is to show that the efficient influence function for $\theta(P)$ in the MSM is characterized up to proportionality as follows:

$$\varphi^{eff}_{msm}(O)\propto s(Z)\left[\frac{I(A=0)\tilde{q}(B|C,L)}{p(A|C,L)p(B|A,C,L)}\{Y-m(A,B,C,L)\}+\Big\{m(0,B^{g^\dagger},C,L)-g(Z;\theta(P))\Big\}\right]. \tag{C.20}$$

Proof. The least false parameter $\theta(P)$ is the least false value that solves equation (C.19). We will use this equation to derive the influence function of $\theta(P)$ in $\mathcal{M}_{np}$. Recall from before that the efficient influence function in the nonparametric model $\mathcal{M}_{np}$ is defined as the unique mean zero, finite variance random variable $\varphi^{\text{eff}}_{msm}(O)$ such that

$$\frac{d\theta(P_\epsilon)}{d\epsilon}\Big|_{\epsilon=0}=E\{\varphi^{\text{eff}}_{msm}(O)S(O)\}.$$

Thus, differentiating (C.19) with respect to ϵ on both sides, we obtain:

$$\begin{aligned}0=&\frac{d}{d\epsilon}E_{P_\epsilon}[s(Z)\{\xi(L;P)-g(Z;\theta(P))\}]\Big|_{\epsilon=0}+\\&\frac{d}{d\epsilon}E_P\Big[s(Z)\{E_P\Big\{m(0,B^{g^\dagger},C,L;P_\epsilon)|L\Big\}-g(Z;\theta(P))\}\Big]\Big|_{\epsilon=0}+\\&\frac{d}{d\epsilon}E_P\Big[s(Z)\{E_{P_\epsilon}\Big\{m(0,B^{g^\dagger},C,L;P)|L\Big\}-g(Z;\theta(P))\}\Big]\Big|_{\epsilon=0}+\end{aligned}$$

$$
\begin{aligned}
&\frac{d}{d\epsilon}E_P[s(Z)\{\xi(L;P)-g(Z;\theta(P_\epsilon))\}]\Big|_{\epsilon=0}\\
=&E_P[s(Z)\{\xi(L;P)-g(Z;\theta(P))\}S(L)]+\\
&E_P\Big\{s(Z)\Big(E_P\Big[E_P\Big\{YS(Y|A{=}0,B^{g^\dagger},C,L)|A{=}0,B^{g^\dagger},C,L\Big\}|L\Big]\Big)\Big\}+\\
&E_P\Big[s(Z)\{E_P\Big\{m(0,B^{g^\dagger},C,L;P)S(A,B,C|L)|L\Big\}\}\Big]+\\
&E_P\Big[-s(Z)\nabla g(Z;\theta(P))\frac{d\theta(P_\epsilon)}{d\epsilon}\Big|_{\epsilon=0}\Big]\\
=&E_P[s(Z)\{\xi(L;P)-g(Z;\theta(P))\}S(O)]\\
&E_P\Big\{s(Z)\frac{I(A{=}0)\tilde{q}(B|C,L)}{p(A,B|C,L)}YS(Y|A,B,C,L)\Big\}+\\
&E_P\Big[s(Z)m(0,B^{g^\dagger},C,L;P)S(A,B,C|L)\Big]+\\
&E_P\Big[-s(Z)\nabla g(Z;\theta(P))\frac{d\theta(P_\epsilon)}{d\epsilon}\Big|_{\epsilon=0}\Big]\\
=&E_P[s(Z)\{\xi(L;P)-g(Z;\theta(P))\}S(O)]+\\
&E_P\Big\{s(Z)\frac{I(A{=}0)\tilde{q}(B|C,L)}{p(A,B|C,L)}\{Y-m(A,B,C,L)\}S(Y|A,B,C,L)\Big\}+\\
&E_P\Big[s(Z)\{m(0,B^{g^\dagger},C,L;P)-\xi(L;P)\}S(A,B,C|L)\Big]-\\
&E_P\Big[s(Z)\nabla g(Z;\theta(P))\frac{d\theta(P_\epsilon)}{d\epsilon}\Big|_{\epsilon=0}\Big]\\
=&E_P\Big[s(Z)\{m(0,B^{g^\dagger},C,L;P)-g(Z;\theta(P))\}S(O)\Big]+\\
&E_P\Big\{s(Z)\frac{I(A{=}0)\tilde{q}(B|C,L)}{p(A,B|C,L)}\{Y-m(A,B,C,L)\}S(O)\Big\}-\\
&E_P\Big[s(Z)\nabla g(Z;\theta(P))\frac{d\theta(P_\epsilon)}{d\epsilon}\Big|_{\epsilon=0}\Big]
\end{aligned}
$$

After rearranging the terms, we obtain the following:

$$
\begin{aligned}
&\frac{d\theta(P_\epsilon)}{d\epsilon}\Big|_{\epsilon=0}=\underbrace{E_P[s(Z)\nabla g(Z;\theta(P))]^{-1}}_{:=\mathbb{C}^{-1}}\times\\
&E_P\left(s(Z)\left[\frac{I(A{=}0)\tilde{q}(B|C,L)}{p(A|C,L)p(B|A,C,L)}\{Y-m(A,B,C,L)\}+\Big\{m(0,B^{g^\dagger},C,L)-g(Z;\theta(P))\Big\}\right]S(O)\right)\\
&=E_P\left(\mathbb{C}^{-1}s(Z)\left[\frac{I(A{=}0)\tilde{q}(B|C,L)}{p(A|C,L)p(B|A,C,L)}\{Y-m(A,B,C,L)\}+\Big\{m(0,B^{g^\dagger},C,L)-g(Z;\theta(P))\Big\}\right]S(O)\right)
\end{aligned}
$$

where we $\mathbb{C}$ denotes a constant square matrix that is assumed to be invertible. □

D. Asymptotic distribution of efficient influence function-based estimators

D.1 Non-MSM estimators In observational studies, model misspecification in the estimation of nuisance functions can induce biased estimates of the ACE. In recent years, there has been an explosion in developing flexible data-adaptive methods (e.g. kernel smoothing, generalized additive models, ensemble learners, random forest) combined with doubly robust estimators that can reduce the risk of model misspecification and provide valid causal inference. These machine learning techniques offer more protection against model misspecification than the parametric models.

From first order expansion of a singly-robust plug-in estimator (IPW and ICE estimators), it can be shown that we require the nuisance parameter estimators to converge to the truth at rate $n^{-1/2}$. However, this is not possible for non-parametric conditional mean functions as this rate is not attainable for these types of functions. However when doubly robust estimators are used with data-adaptive methods this issue largely disappears are doubly robust estimators enjoy the small bias property (Newey et al., 2004).

In this section we will examine the Remainder or Bias term from the following decomposition. For notational brevity, we suppress O in the equations below. For generality, suppose that $\mu^g(\hat{P})$ is an estimator that solves the estimating equations based on the efficient influence function. We have that

$$\begin{aligned}
\sqrt{n}(\mu^g(\hat{P})-\mu^g(P)) &= \sqrt{n}\left[\mathbb{P}_n(\varphi^{eff}(\hat{P}))-P(\varphi^{eff}(\hat{P}))\right]+\sqrt{n}\left[\mu^g(\hat{P})+P(\varphi^{eff}(\hat{P}))-\mu^g(P)\right] \\
&= \mathbb{G}_n(\varphi^{eff}(P))+\mathbb{G}_n[\varphi^{eff}(\hat{P})-\varphi^{eff}(P)]+ \\
&\quad \sqrt{n}\left[\mu^g(\hat{P})+P(\varphi^{eff}(\hat{P}))-\mu^g(P)\right] \\
&= \underbrace{\mathbb{G}_n(\varphi(P))}_{T_1}+\underbrace{\mathbb{G}_n[\varphi(\hat{P})-\varphi(P)]}_{T_2}+ \\
&\quad \sqrt{n}\left[\underbrace{\mu^g(\hat{P})+P(\varphi^{eff}(\hat{P}))-\mu^g(P)}_{R}\right]
\end{aligned}$$

where $\mathbb{G}_n[X]=\sqrt{n}(\mathbb{P}_n-P)(X)$ for any X and we define $\varphi(O;\tilde{P})=\varphi^{eff}(O;\tilde{P})+\mu^g(O;\tilde{P})$ for any $\tilde{P}$. The first term given by T_1 is a centered sample average which converges to a mean zero Normal distribution by the central limit theorem. The second term is known as an empirical process term, which can be shown to be $o_p(1)$ under Stochastic Equicontinuity if we assume that nuisance functions and their corresponding estimators are not too complex and belong to Donsker class (by Lemma 19.24 of Van Der Vaart, 2000) . Alternatively, one can use sample splitting and cross fitting to overcome issues with overfitting (Chernozhukov et al., 2018).

We will denote nuisance functions by $\eta := (\eta_1(P), \eta_2(P)) = (P(A,B|C,L), m(A,B,C,L))$ and the corresponding nuisance function estimates as $\hat{\eta} := (\eta_1(\hat{P}), \eta_2(\hat{P})) = (\hat{P}(A,B| C,L), \hat{m}(A,B,C,L))$. Formally, we assume the following conditions:

C1. $\eta_1(\hat{P}) - \eta_1(P) = o_p(1)$ and $\eta_2(\hat{P}) - \eta_2(P) = o_p(1)$.

C2. $E_P\left[\varphi^{\text{eff}}(O)^2\right] < \infty$

C3. $\varphi^{\text{eff}}(O)$ and $\hat{\varphi}^{\text{eff}}(O)$ belong to a Donsker family.

C4. $\|\hat{\varphi}^{\text{eff}}(O) - \varphi^{\text{eff}}(O)\|_2^2 \xrightarrow{p} 0$

where $\hat{\varphi}^{\text{eff}}(O)$ denotes an estimator of $\varphi^{\text{eff}}(O)$ where all nuisance functions estimators are exactly the same as those in the TMLE estimator. It is not hard to show that $\mathbb{P}_n\{\hat{\varphi}^{\text{eff}}(O)\} = 0$ by construction.

The last term is known as the remainder or bias term. We will need to show that $\sqrt{n}R = o_p(1)$ under some conditions about the convergence rates of the nuisance functions. As before, we let $r(A,B,C,L) = \frac{I(A=0)\tilde{q}(B|C,L)}{p(A,B|C,L)}$ and $\hat{r}(A,B,C,L) = \frac{I(A=0)\hat{\tilde{q}}(B|C,L)}{\hat{p}(A,B|C,L)}$:

$$
\begin{aligned}
&\mu^g(\hat{P}) + P(\varphi^{eff}(\hat{P})) - \mu^g(P)\\
&= E_P\left[\hat{r}(A,B,C,L)\{Y - \hat{m}(A,B,C,L)\} + \hat{m}(0,B^{g^\dagger},C,L) - \mu^g(P)\right]\\
&= E_P\left[\hat{r}(A,B,C,L)\{Y - \hat{m}(A,B,C,L)\} + \hat{m}(0,B^{g^\dagger},C,L) - m(0,B^{g^\dagger},C,L)\right]\\
&= E_P\left[\hat{r}(A,B,C,L)\{m(A,B,C,L) - \hat{m}(A,B,C,L)\} + \hat{m}(0,B^{g^\dagger},C,L) - m(0,B^{g^\dagger},C,L)\right]
\end{aligned}
$$

Noting that $E_P[m(0,B^{g^\dagger},C,L)] = E_P[r(A,B,C,L)m(A,B,C,L)]$ and $E_P[\hat{m}(0,B^{g^\dagger},C,L)] = E_P[r(A,B,C,L)\hat{m}(A,B,C,L)]$, it follows that:

$$
\begin{aligned}
&\mu^g(\hat{P}) + P(\varphi^{eff}(\hat{P})) - \mu^g(P)\\
&= E_P[\hat{r}(A,B,C,L)\{m(A,B,C,L) - \hat{m}(A,B,C,L)\} + r(A,B,C,L)\{\hat{m}(A,B,C,L) - m(A,B,C,L)\}]\\
&= E_P[\{\hat{r}(A,B,C,L) - r(A,B,C,L)\}\{m(A,B,C,L) - \hat{m}(A,B,C,L)\}]
\end{aligned}
$$

By an application of Cauchy-Schwartz, we can show that as long as:

$$\|\hat{r}(A,B,C,L) - r(A,B,C,L)\| \; \|\hat{m}(A,B,C,L) - m(A,B,C,L)\| = O_p(n^{-\nu}),$$

for $\nu > 1/2$ and where $\|f(x)\| = \left\{\int |f(x)|^2 dP(x)\right\}^{1/2}$, i.e. the $L_2(P)$ norm. Then, $\sqrt{n}\left\{\mu^g(\hat{P}) + P(\varphi^{eff}(\hat{P})) - \mu^g(P)\right\} = o_p(1)$. This can be accomplished, for example, if the nuisance functions are each consistently estimated at a rate faster than $n^{-1/4}$.

D.2 MSM estimators As before, we let $\xi(L;P)=E_P\left\{m(0,B^{g^\dagger},C,L)\,|\,L\right\}=E(Y^g\,|\,L)$ and $g(Z;\theta):=\text{expit}(s(Z)^T\theta)$. As shown in Web Appendix C, the efficient influence function for $\theta(P)$ in the MSM is characterized up to proportionality is given as follows:

$$\varphi^{eff}_{msm}(O)\propto s(Z)\left[\frac{I(A=0)\tilde{q}(B|C,L)}{p(A|C,L)p(B|A,C,L)}\{Y-m(A,B,C,L)\}+\left\{m(0,B^{g^\dagger},C,L)-g(Z;\theta(P))\right\}\right].$$

We will denote nuisance functions by $\eta:=(\eta_1(P),\eta_2(P))=(P(A,B|C,L),m(A,B,C,L))$ and the corresponding nuisance function estimates as $\hat{\eta}:=(\eta_1(\hat{P}),\eta_2(\hat{P}))=(\hat{P}(A,B|C,L),\hat{m}(A,B,C,L))$. For ease of notation, we also let $\theta:=\theta(P)$, and we let

$$\phi(O;\eta,\theta):=s(Z)\left[\frac{I(A=0)\tilde{q}(B|C,L)}{p(A|C,L)p(B|A,C,L)}\{Y-m(A,B,C,L)\}+\left\{m(0,B^{g^\dagger},C,L)-g(Z;\theta(P))\right\}\right].$$

Formally, we assume the following conditions throughout:

C1. $\|\hat{\theta}-\theta\|=o_p(1)$, $\eta_1(\hat{P})-\eta_1(P)=o_p(1)$ and $\eta_2(\hat{P})-\eta_2(P)=o_p(1)$.

C2. $\mathbb{C}:=E_P[s(Z)\nabla g(Z;\theta(P))]$ is nonsingular.

C3. $E_P[\phi(O;\eta,\theta)^2]<\infty$

C4. $\phi(O;\eta,\theta)$ and $\hat{\phi}(O;\eta,\theta)$ belong to a Donsker family.

C5. $\|\hat{\phi}(O;\eta,\theta)-\phi(O;\eta,\theta)\|_2^2\xrightarrow{p}0$

Theorem 4 states that under the aforementioned conditions, and further suppose that the product mean-squared error condition of Theorem 2 holds, then it follows that:

$$\sqrt{n}\left(\hat{\theta}-\theta\right)\rightsquigarrow MVN\left(0,\mathbb{C}^{-1}\text{Cov}\{\phi(O;\eta,\theta)\}C^{-1^T}\right).$$

Proof. By construction, we solve for θ in the following estimating equation:

$$\mathbb{P}_n[\phi(O;\hat{\eta};\theta)]=0.$$

Suppose that $\hat{\theta}:=\theta(\hat{P})$ solves the aforementioned estimating equation. Thus, by expanding the estimating equation around $\theta:=\theta(P)$ (with a slight abuse of notation), we obtain the following:

$$\mathbb{P}_n\left[\phi(O;\hat{\eta};\hat{\theta}\right]=\mathbb{P}_n[\phi(O;\hat{\eta};\theta)]+\mathbb{P}_n\left[\phi'(O;\hat{\eta};\tilde{\theta})\right](\hat{\theta}-\theta)$$

where $\tilde{\theta}$ is an intermediate value between $\hat{\theta}$ and θ, and $\phi'(O;\hat{\eta};\tilde{\theta}) = \frac{d\phi(O;\hat{\eta};\tilde{\theta})}{d\theta}$. After rearranging the terms in the previous and multiplying the left-hand-side and right-hand-side by $\sqrt{n}$, we obtain:

$$\sqrt{n}(\hat{\theta}-\theta) = -\left(\mathbb{P}_n\left[\phi'(O;\hat{\eta};\tilde{\theta})\right]\right)^{-1}\sqrt{n}\mathbb{P}_n[\phi(O;\hat{\eta};\theta)] \tag{D.21}$$

Note that by the Uniform Law of Large Numbers and by the Continuous Mapping Theorem, $\mathbb{P}_n\left[\phi'(O;\hat{\eta};\tilde{\theta})\right] \xrightarrow{p} E_P[s(Z)\nabla g(Z;\theta(P))]$ (defined as $\mathbb{C}$ previously), provided that condition C2. holds. We will utilize this result later.

Next, we look at the term $\sqrt{n}\mathbb{P}_n[\phi(O;\hat{\eta};\theta)]$ in (D.21):

$$\begin{aligned}\sqrt{n}\mathbb{P}_n[\phi(O;\hat{\eta};\theta)] &= \sqrt{n}(\mathbb{P}_n[\phi(O;\hat{\eta};\theta)] - \mathbb{P}[\phi(O;\eta;\theta)]) \\ &= \underbrace{\mathbb{G}_n[\phi(O;\eta,\theta)]}_{T_1} + \underbrace{\mathbb{G}_n[\phi(O;\hat{\eta},\theta)-\phi(O;\eta,\theta)]}_{T_2} + \underbrace{\sqrt{n}P[\phi(O;\hat{\eta},\theta)-\phi(O;\eta,\theta)]}_{R}\end{aligned}$$

Again, the first term given by T_1 is a centered sample average which, under conditions C3. converges to a mean zero Multivariate Normal distribution by the central limit theorem such that $\mathbb{G}_n[\phi(O;\eta,\theta)] \rightsquigarrow MVN(0, \mathrm{Cov}\{\phi(O;\eta,\theta)\})$. The second term T_2 is an empirical process term, which can be shown to be $o_p(1)$ under Stochastic Equicontinuity if we assume that nuisance functions and their corresponding estimators are not too complex and belong to Donsker class (assuming conditions C4. and C5.; by Lemma 19.24 of Van Der Vaart, 2000). Alternatively, one can use sample splitting and cross fitting to overcome issues with overfitting (Chernozhukov et al., 2018). Then the results follow once we show that the remainder term $\sqrt{n}R$ is $o_p(1)$. This can be accomplished, for example, if $r(A,B,C,L)$ and $m(A,B,C,L)$ functions are consistently estimated at a rate faster than $n^{-1/4}$, and the proof follows analogously to that of Non-MSM estimators in Web Appendix D.1. Thus, putting it all together, it follows then in:

$$\sqrt{n}(\hat{\theta}-\theta) = -\left(\mathbb{P}_n\left[\phi'(O;\hat{\eta};\tilde{\theta})\right]\right)^{-1}\sqrt{n}\mathbb{P}_n[\phi(O;\hat{\eta};\theta)]$$

we have that $\mathbb{P}_n\left[\phi'(O;\hat{\eta};\tilde{\theta})\right] \xrightarrow{p} \mathbb{C}$, and $\sqrt{n}\mathbb{P}_n[\phi(O;\hat{\eta};\theta)] \rightsquigarrow MVN(0, \mathrm{Cov}\{\phi(O;\eta,\theta)\})$. By Slutsky's theorem, it follows then:

$$\sqrt{n}\left(\hat{\theta}-\theta\right) \rightsquigarrow MVN\left(0, \mathbb{C}^{-1}\mathrm{Cov}\{\phi(O;\eta,\theta)\}C^{-1^T}\right).$$

In particular for $k=0,\ldots,p$,

$$\sqrt{n}\left(\hat{\theta}_k-\theta_k\right) \rightsquigarrow N\left(0, \left[\mathbb{C}^{-1}\mathrm{Cov}\{\phi(O;\eta,\theta)\}C^{-1^T}\right]_{kk}\right),$$

where $\left[\mathbb{C}^{-1}\text{Cov}\{\phi(O;\eta,\theta)\}C^{-1^T}\right]_{kk}$ denotes the kth diagonal element of the variance-covariance matrix. $\square$

E. Other MSM estimators

The MSM outcome regression estimator generalizes the previous marginal outcome regression estimator. It entails first estimating $\hat{m}(0,B^{g^\dagger},C,L)$ for $m(0,B^{g^\dagger},C,L)$ and subsequently solving for θ parameter in the following set of estimating equations:

$$\mathbb{P}_n\left(s(Z)\left[\hat{m}(0,B^{g^\dagger},C,L)-g(Z;\theta)\right]\right)=0,$$

where $g(Z;\theta)=\text{expit}\{s(Z)^T\theta\}$.

Another estimator is the MSM IPW estimator that entails solving for θ in the following set of estimating equations:

$$\mathbb{P}_n\left(s(Z)\hat{W}[Y-g(Z;\theta)]\right)=0,$$

where as before

$$\hat{W}=\frac{I(A=0)\hat{\tilde{q}}(B|C,L)}{\hat{\lambda}_A(C,L)\hat{\lambda}_B(A,C,L)}$$

and

$$\hat{\tilde{q}}(B|C,L)=\left[\sum_a\left\{I(a\leq B\leq x)\hat{\lambda}_{B-a}(a,C,L)+I(B=x)\left[1-\sum_{b=0}^{x-a}\hat{\lambda}_b(A,C,L)\right]\right\}\hat{\lambda}_a(C,L)\right].$$

F. Additional simulation results and additional simulation study using Highly Adaptive Lasso (HAL) (van der Laan, 2017)

F.1 Detailed simulation results from main text

[Table 1 about here.]

F.2 Additional simulation studies We conduct additional simulation studies to compare the performance of algorithms that use machine learning to estimate the nuisance functions for the parameters in an MSM. Specifically, we compare the proposed TMLE with IPW and ORE.

We simulated 1000 hypothetical cohorts of $n=(500,1000,5000)$ comprising the following variables: (U,L_1,L_2,A,B,C,Y), where $U\sim\text{Unif}[0,1]$ denotes an unmeasured baseline covariate, $L_1\sim\text{Ber}(0.5)$ and $L_2\sim\text{Unif}[0,1]$ denotes a measured baseline covariates, $(A,B,C)^T$ denotes a vector of exposures where $A\sim\text{Bin}(3,\text{expit}(-2+0.75L_1+0.5L_2+U))$, $B\sim\text{Bin}(3,\text{expit}(-1+0.5L_1-0.2\exp(0.5L_2)+U))$, $C\sim\text{Bin}(3,\text{expit}(-1+L_1+U))$, and $Y\sim\text{Ber}(\text{expit}(-1-A+B+C-2L_1+L_2-0.25L_2^2+0.25L_1L_2))$ denotes a binary outcome. We consider estimating the parameters in the MSM given by $E(Y^g|L;\theta)=\text{expit}(\theta_0+\theta_1L_2)$.

Since HAL can only handle `binomial`, `gaussian`, `poisson`, `cox` or `mgaussian` family objects, we will need to convert our multinomial exposure variables (e.g., for A, we have the $\text{supp}(A)=\{0,1,2,3\}$) to binary outcomes and estimating $P(A=k|L)$ for $k\geq 0$. This can be done by generating a series of logistic regression models as follows:

(1) Calculate $\hat{P}(A=0|L)$ and $\hat{P}(A\neq 0|L)=\hat{P}(A>0|L)$ using a logistic regression model for binary events $\{A=0\}$ and $\{A\neq 0\}$.
(2) For $k=1,2,...$ calculate $\hat{P}(A=k|A>k-1,L)$ and $\hat{P}(A\neq k|A>k-1,L)=\hat{P}(A>k|A>k-1,L)$ using logistic regression models.
(3) Using the fact that $P(A=k|L)=\prod_{j=0}^{k}P(A=j|A>j-1,L)$ and calculate $\hat{P}(A=k|L)=\prod_{j=0}^{k}\hat{P}(A=j|A>j-1,L)$ using the fitted logistic regression models from previous steps. For instance, for $k=2$, we can calculate $\hat{P}(A=2|L)=P(A=2|A>1,L)P(A=1|A>0,L)P(A=0|L)$.

Equivalent methods can also be applied to B where $\text{supp}(B)=\{0,1,2,3\}$.

Table G.2 compares the performance of the 3 estimators, and the results are similar to those in the main manuscript. The ORE and IPW estimators show more bias compared with the TMLE as they are not expected to converge at $\sqrt{n}$ rates when machine learning is used for nuisance parameter estimation. TMLE, on the other hand, show little to no bias in all instances. This agrees with theory as TMLE allows

the nuisance functions to converge at slower nonparametric rates. The estimated coverage probability of the confidence intervals for TMLE based on the asymptotic variance gets closer to the nominal 95% as sample size increases. For ($\hat{\beta}_0$,$\hat{\beta}_1$), the 95% coverage probability is [(91.6,90.4), (92.2,92.6), (94.3,93.7)] for n=(500, 1000, 5000), respectively.

[Table 2 about here.]

G. Additional sensitivity analysis of Nurses' Health Study

Under no diet intervention, the estimated two-year and four-year mortality risk are 1.97% (95% CI: 1.82% – 2.11%) and 5.37% (95% CI: 5.14%–5.60%), respectively. We estimated the two-year and four-year mortality risk under the dietary substitution intervention to be 1.60% (95% CI: 1.26% – 1.91%) and 4.43% (95% CI: 3.86% – 5.03%) using TMLE with parametric models, and 1.68% (95% CI: 1.42% – 1.94%) and 4.72% (95% CI: 4.12% – 5.25%) using TMLE with HAL, respectively. The estimates from both of the methods are similar (see Figure 6). Based on the results from TMLE with HAL, our estimates suggest that the intervention would slightly decrease the two-year mortality by an estimated 0.29% (95% CI: 0.06%–0.52%) and the four-year mortality by an estimated 0.65% (95% CI: 0.16%–1.15%).

Sensitivity analysis was performed using various values of x={8, 9, 10}, and the results closely resemble those obtained when x=7 (see Figures G.3 and G.3). That is, there is some evidence to suggest that the proposed substitution strategy would slightly decrease the two-year and four-year mortality rate.

[Figure 2 about here.]

[Figure 3 about here.]

References

Chernozhukov, V., Chetverikov, D., Demirer, M., Duflo, E., Hansen, C., Newey, W., and Robins, J. (2018). Double/debiased machine learning for treatment and structural parameters. *The Econometrics Journal*, 21:C1–C68.

Newey, W. K., Hsieh, F., and Robins, J. M. (2004). Twicing kernels and a small bias property of semiparametric estimators. *Econometrica*, 72:947–962.

Petersen, M., Schwab, J., Gruber, S., Blaser, N., Schomaker, M., and van der Laan, M. (2014). Targeted maximum likelihood estimation for dynamic and static longitudinal marginal structural working models. *Journal of causal inference*, 2:147–185.

van der Laan, M. (2017). A generally efficient targeted minimum loss based estimator based on the highly adaptive lasso. *The international journal of biostatistics*, 13(2).

Van Der Vaart, A. W. (2000). *Asymptotic statistics*, volume 3. Cambridge university press.

Zheng, W., Petersen, M., and van der Laan, M. J. (2016). Doubly robust and efficient estimation of marginal structural models for the hazard function. *The international journal of biostatistics*, 12(1):233–252.

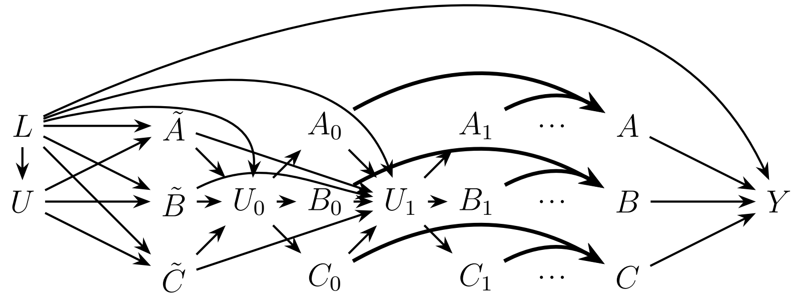

Figure G.1. Directed Acyclic Graph (DAG) depicting underlying data structure of the observational data. Here, $\tilde{A}$, $\tilde{B}$, and $\tilde{C}$ denote the planned weekly portions of food intake in groups $\mathcal{A}$, $\mathcal{B}$ and $\mathcal{C}$; A, B, and C denote the actual weekly portions of food intake in groups $\mathcal{A}$, $\mathcal{B}$ and $\mathcal{C}$ that will be intervened on; Y a binary outcome; L a set of potential confounders measured at baseline that affect planned dietary intake and outcome; and U a set of potential baseline common causes of planned dietary intake decisions. (A_k,B_k,C_k) denote the intake of $\mathcal{A}$, $\mathcal{B}$ and $\mathcal{C}$ in time interval k (k=0,...,K), respectively, and U_k denotes underlying factors that affect (A_k,B_k,C_k).

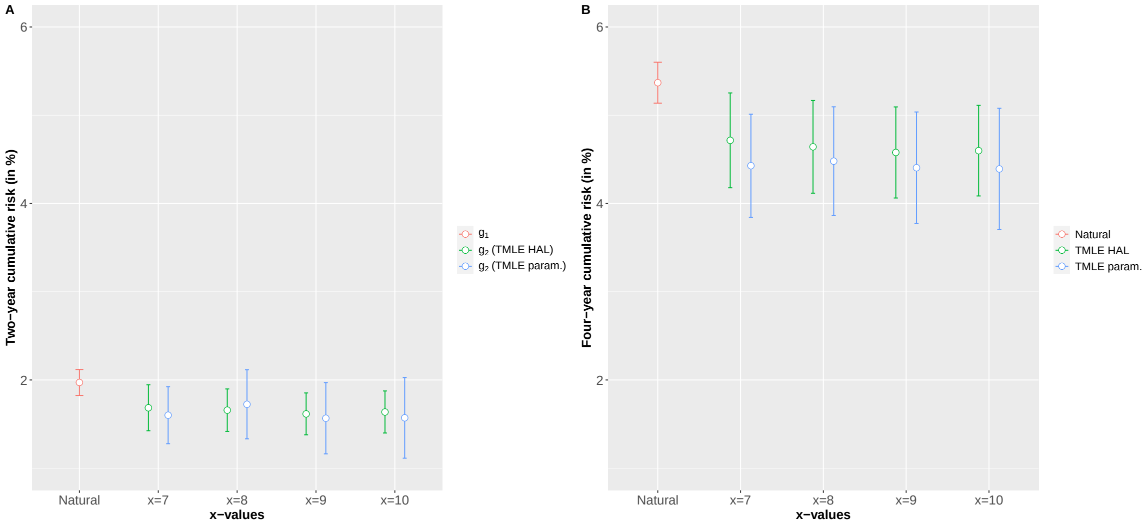

FIGURE G.2. Estimated two-year and four-year cumulative risk for x=$\{7,8,9,10\}$ using TMLE with parametric models for estimating nuisance functions and TMLE with HAL for estimating nuisance functions.

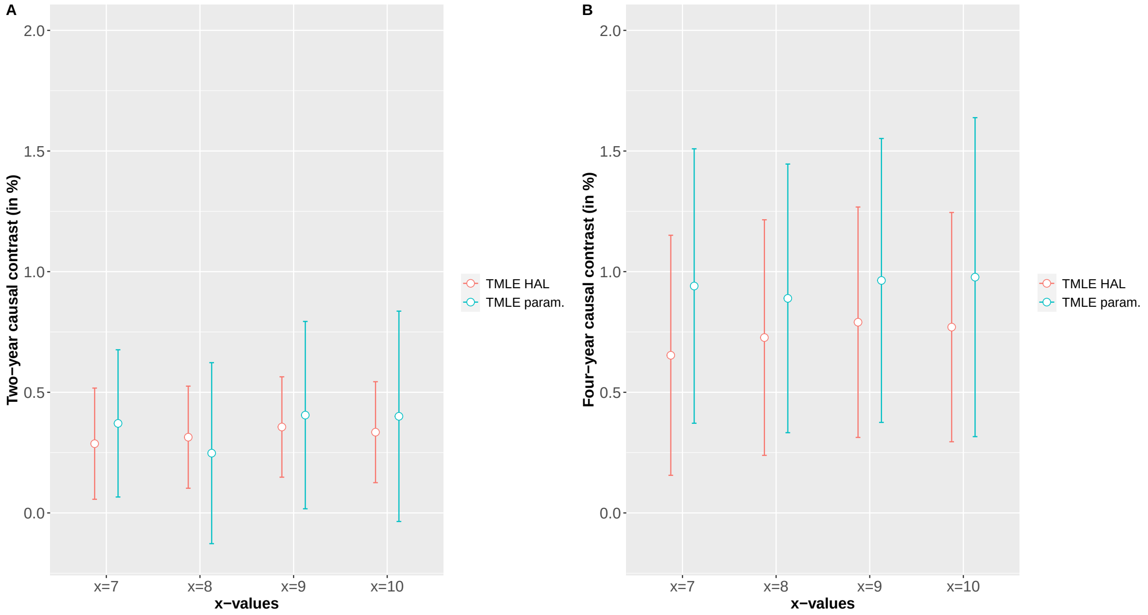

FIGURE G.3. Estimated two-year and four-year causal contrast for $x = \{7,8,9,10\}$ using TMLE with parametric models for estimating nuisance functions and TMLE with HAL for estimating nuisance functions.

	(i) Correctly Specified				(ii) Misspecified outcome model				(iii) Misspecified exposure model			
ORE	Bias	SE	MSE	CP	Bias	SE	MSE	CP	Bias	SE	MSE	CP
θ_0	0.003	0.065	0.004	95.6	−0.861	0.051	0.745	0.00	0.003	0.065	0.004	95.6
θ_1	−0.002	0.076	0.006	94.5	0.734	0.088	0.546	0.00	−0.002	0.076	0.006	94.5
IPW	Bias	SE	MSE	CP	Bias	SE	MSE	CP	Bias	SE	MSE	CP
θ_0	0.006	0.090	0.008	95.1	0.006	0.090	0.008	95.1	0.154	0.090	0.032	64.2
θ_1	−0.003	0.175	0.031	92.2	−0.003	0.175	0.031	92.2	−0.568	0.131	0.340	0.50
TMLE	Bias	SE	MSE	CP	Bias	SE	MSE	CP	Bias	SE	MSE	CP
θ_0	0.006	0.090	0.008	95.1	0.006	0.090	0.008	95.1	0.006	0.090	0.008	95.5
θ_1	−0.006	0.175	0.031	92.2	0.000	0.175	0.031	92.3	−0.009	0.122	0.015	95.1

Table G.1. Results for simulation study for n=5000 using parametric models for nuisance functions: Bias, standard error (SE), mean squared error (MSE) and 95% coverage probability (CP; ×100). True value of $(\theta_0,\theta_1)=(2.347,-1.635)$.

Table G.2. Results for simulation study for n=(500,1000,5000) using nonparametric models for nuisance functions: Bias, standard error (SE), and mean squared error (MSE). True value of (θ_0,θ_1)= (0.996,0.822).

	n=500			n=1000			n=5000		
ORE	Bias	SE	MSE	Bias	SE	MSE	Bias	SE	MSE
θ_0	-0.085	0.256	0.075	-0.044	0.178	0.042	-0.024	0.086	0.020
θ_1	-0.062	0.398	0.188	-0.044	0.293	0.109	-0.011	0.141	0.036
IPW	Bias	SE	MSE	Bias	SE	MSE	Bias	SE	MSE
θ_0	0.049	0.562	0.344	0.050	0.384	0.179	0.037	0.171	0.051
θ_1	-0.069	1.093	1.226	-0.076	0.756	0.596	-0.080	0.342	0.154
TMLE	Bias	SE	MSE	Bias	SE	MSE	Bias	SE	MSE
θ_0	0.003	0.474	0.242	0.001	0.337	0.133	-0.006	0.156	0.038
θ_1	0.045	0.889	0.800	0.029	0.665	0.446	0.016	0.312	0.112
